# Heterochromatin formation in *Drosophila* requires genome-wide histone deacetylation in cleavage chromatin before mid-blastula transition in early embryogenesis

**DOI:** 10.1007/s00412-020-00732-x

**Published:** 2020-01-16

**Authors:** Matthias Walther, Sandy Schrahn, Veiko Krauss, Sandro Lein, Jeannette Kessler, Thomas Jenuwein, Gunter Reuter

**Affiliations:** 1 Developmental Genetics, Institute of Biology, Martin Luther University Halle, Weinbergweg 10, 06120 Halle/S., Germany; 2 Max Planck Institute of Immunobiology and Epigenetics, Stübeweg 51, 79108 Freiburg, Germany; 3 Cluster of Excellence in Plant Science (CEPLAS), University of Cologne, Biocenter, 50674 Cologne, Germany

**Keywords:** Heterochromatin, Histone deacetylation, Mid-blastula transition, *Drosophila melanogaster*

## Abstract

*Su(var)* mutations define epigenetic factors controlling heterochromatin formation and gene silencing in *Drosophila*. Here, we identify SU(VAR)2-1 as a novel chromatin regulator that directs global histone deacetylation during the transition of cleavage chromatin into somatic blastoderm chromatin in early embryogenesis. SU(VAR)2-1 is heterochromatin-associated in blastoderm nuclei but not in later stages of development. In larval polytene chromosomes, SU(VAR)2-1 is a band-specific protein. SU(VAR)2-1 directs global histone deacetylation by recruiting the histone deacetylase RPD3. In *Su(var)2-1* mutants H3K9, H3K27, H4K8 and H4K16 acetylation shows elevated levels genome-wide and heterochromatin displays aberrant histone hyper-acetylation. Whereas H3K9me2- and HP1a-binding appears unaltered, the heterochromatin-specific H3K9me2S10ph composite mark is impaired in heterochromatic chromocenters of larval salivary polytene chromosomes. SU(VAR)2-1 contains an NRF1/EWG domain and a C2HC zinc-finger motif. Our study identifies SU(VAR)2-1 as a dosage-dependent, heterochromatin-initiating SU(VAR) factor, where the SU(VAR)2-1-mediated control of genome-wide histone deacetylation after cleavage and before mid-blastula transition (pre-MBT) is required to enable heterochromatin formation.

**Electronic supplementary material:**

The online version of this article (10.1007/s00412-020-00732-x) contains supplementary material, which is available to authorized users.

## Introduction

The stochastic silencing of a gene when juxtaposed to heterochromatic regions by rearrangements or transposition in position-effect variegation (PEV) has been successfully used in *Drosophila* to reveal epigenetic factors that favor the establishment of either euchromatic or heterochromatic domains (for a review see Girton and Johansen 2008; Elgin and Reuter 2013). Classical genetic screens in *Drosophila* for modifiers of PEV estimate that about 200 independent loci enhance or suppress PEV, the so-called *E(var)* and *Su(var)* genes. The few molecularly defined E(VAR) proteins exert their function mainly at euchromatic regions (Farkas et al. 1994; DeRubertis et al. 1996; Dorn et al. 1993a; Weiler 2007; Lloret-Llinares et al. 2008). In contrast, SU(VAR) factors stabilize the repressed chromatin state and are thus often associated with heterochromatic regions of *Drosophila* (Elgin and Reuter 2013). Of the estimated 100 *Su(var)* loci, only about 20% have so far been defined by positional cloning or candidate gene analysis. Amongst those are several prominent factors in the establishment and maintenance of heterochromatin, e.g. the H3K9 methyltransferase (KMTase) SU(VAR)3-9 (Tschiersch et al. 1994; Rea et al. 2000; Schotta et al. 2002), the H3K9me2/3-binding protein SU(VAR)2-5 (HP1a) (Eissenberg et al. 1992; Lachner et al. 2001; Fischle et al. 2003) and the H3K4 demethylase SU(VAR)3-3 (LSD1) (Rudolph et al. 2007; Di Stefano et al. 2007). In addition, mutations in a number of chromatin regulators, including the H4K20 KMTase Suv4-20 (Schotta et al. 2004), the Jumonji C domain-containing protein Jarid2/LID (Sasai et al. 2007), or the protein phosphatase PP1 (Baksa et al. 1993), modify PEV variegation, indicating a role in heterochromatin formation. Although most of these are SU(VAR) factors, genetic analysis suggests an equal number of *Su(var)* and *E(var)* genes (Dorn et al. 1993b). Combined, these studies revealed the molecular identity of about 40 chromatin factors in *Drosophila*, many of which are conserved in the mammalian system (Fodor et al. 2010). Thus, the identification of novel *Su(var)* genes has far-reaching implications in providing insight into the molecular basis of *Drosophila* heterochromatin, and indicates that many of the newly characterized pathways might also operate in other eukaryotes (Grewal and Jia 2007; Allshire and Madhani 2017).

In addition to their role in constitutive heterochromatin, many SU(VAR) factors have functions in other chromatin-dependent processes such as genome stability (Janssen et al. 2018), reprogramming/pluripotency (Soufi et al. 2012; Lu et al. 2014), transposon silencing (Karimi et al. 2011; Bulut-Karslioglu et al. 2013) and epithelial-mesenchymal transition in (EMT)/tumor progression (Ting et al. 2011; Millanes-Romero et al. 2013). Thus, the identification of novel *Su(var)* genes has the potential not only to provide further mechanistic insights into the epigenetic roles of SU(VAR) factors, but also to reveal the molecular pathways underpinning new functions of heterochromatin.

Here, we describe a novel SU(VAR) factor with a fundamental role in heterochromatin formation during *Drosophila* development. The *Su(var)2-1* gene encodes a NRF1-domain protein with differential chromatin association throughout development. It is heterochromatin-associated in early blastoderm but later in development, it is an abundant band protein. SU(VAR)2-1 controls development-specific histone deacetylation at pre-mid-blastula by recruiting the RPD3 (HDAC1) histone deacetylase. Furthermore, SU(VAR)2-1 is required for establishment of the heterochromatin-specific H3K9me2S10phos double histone modification mark. The SU(VAR)2-1 protein has a crucial role in global chromatin reorganization at pre-MBT by controlling genome-wide histone deacetylation maternally, preceding differential establishment of euchromatic and heterochromatic chromatin domains. SU(VAR)2-1 is thus the first factor to be identified, which is involved in epigenetic processes of chromatin transition after cleavage. This discovery will facilitate analysis of the so far uncharacterized epigenetic processes preceding differentiation of alternative chromatin states in the blastoderm.

## Materials and methods

### *Drosophila* culture, stocks and genetic analysis

Flies were reared on *Drosophila* standard medium at 25 °C. Chromosomes and mutations not noted here are described in FlyBase (http://flybase.org). The *In(1)w*^*m4h*^ rearrangement was used for the analysis of PEV. For P element–mediated transformation, we used the *w*^*1118*^ strain from the Bloomington *Drosophila* Stock Center.

**Fig. 1:**
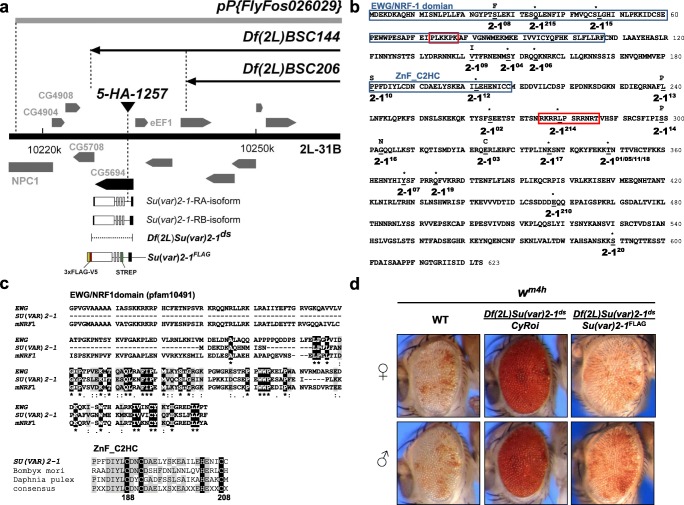
*Su(var)2-1* encodes a NRF domain protein with a C2HC zinc-finger motif. **a** Cytogenetic mapping of *Su(var)2-1* within region 31B in chromosome arm 2L between the distal breakpoints of *Df(2L)BSC144* and *Df(2L)BSC206*. The *pP{RS5}5-HA-1257* element inserted within the first intron of *CG5694* and a CRISPR/Cas9 induced deletion of *CG5694* {*Df(2L)Su(var)2-1*^*ds*^} are allelic to *Su(var)2-1* mutations. The *pP*{*FlyFos026029*} and *P*{*UAST-attB Strep-Su(var)2-1-V5-3xFLAG*} transgenes rescue *Su(var)2-1* mutations. **b** Molecularly defined *Su(var)2-1* mutations including in total 15 stop or frameshift mutations (*) and 7 point mutations. The *Su(var)2-1* alleles *2-1*^*210*^, *2-1*^*214*^ and *2-1*^*215*^ were isolated by Sinclair et al. 1992. The SU(VAR)2-1 protein contains two putative nuclear localization signals (red boxes). **c** In the SU(VAR)2-1N-terminus about 100 amino acids show homology to the C-terminal half of the NRF1/EWG domain of *Drosophila* ERECT WING (EWG) and mammalian NRF1 proteins. In addition, SU(VAR)2-1 contains a C2HC motif between amino acids 188–208. **d** Phenotypic rescue of *Su(var)2-1* mutants by *P*{*UAST-attB Strep-Su(var)2-1-V5-3xFLAG*} expressing a fusion protein with a N-terminal STREP and C-terminal V5-3xFLAG tag under the endogenous *Su(var)2-1* promoter (Abbreviated *Su(var)2-1*^FLAG^)

The 20 *Su(var)2-1* mutants (Fig. 1 and Supplementary Table S1) were isolated by their strong dominant suppressor effect on *white* gene silencing in the sensitized *E(var)* background of *In(1)w*^*m4*^; *T(2;3)ap*^*Xa*^ *+ In(2 L)Cy, ap*^*Xa*^*Cy E(var)3-1*^*01*^ after EMS (2.5 mM) mutagenesis (Reuter et al. 1986). The *Su(var)2-1* alleles *2-1*^*210*^, *2-1*^*214*^ and *2-1*^*215*^ were isolated by Sinclair et al. (1992).

Deficiencies *Df(2L)BSC144* and *Df(2L)BSC206* were obtained from the Bloomington *Drosophila* Stock Center and deficiencies *Df(2L)ED721* and *Df(2L)ED729* were generated according to the method described in Ryder et al. (2007). In *Df(2L)Su(var)2-1*^*ds*^*,* a knock-out of *Su(var)2-1* was generated by the Cas9/sgRNA system according to the method described by Gratz et al. (2013). The *P*{*Sgs3*-*GAL4*} salivary gland cell-specific *GAL4* driver was obtained from the Bloomington *Drosophila* Stock Center.

The transgenes *P*{*w*^*+*^*UAST*-*attB*-*Strep*-*Su(var)2-1*-*V5*-3x*FLAG*}, *P*{*FlyFos026029*-*Su(var)2-1*-*V5*-3x*FLAG*} and *P*{*w*^*+*^*UAS*-*Su(var)2-1*-*EGFP*} were generated for *Su(var)2-1* mutant rescue and expression of SU(VAR)2-1 fusion proteins containing antibody tags (Supplementary Table S1). The *P*{*w*^*+*^*UAST*-*attB*-*Strep*-*Su(var)2-1*-*V5*-3x*FLAG*} and *P*{*FlyFos026029*-*Su(var)2-1*-*V5*-3x*FLAG*} rescue transgenes express SU(VAR)2-1 under the control of the endogenous *Su(var)2-1* promoter and were generated according to the method described by Ejsmont et al. (2009) and Bischof et al. (2007). pP{w^+^ UAST-attB-Strep-Su(var)2-1-V5-3xFLAG} was injected into *attP-ZH-51D* and pP{FlyFos026029-Su(var)2-1-V5-3xFLAG} into *attP2* embryos. The *attP-ZH-51D* [#24483] and *attP2* [#8622] fly lines were received from the Bloomington *Drosophila* Stock Center. In *P*{*w*^*+*^*UAS*-*Su(var)2-1*-*EGFP*} the coding sequence of *Su(var)2-1*-*EGFP* was placed under the control of the *UAS* promoter.

### Molecular cloning and transformation of wild-type rescue constructs

The genomic full-length wild-type *Su(var)2-1* gene was amplified with primers GGGGACAAGTTTGTACAAAAAAGCAGGCTCAAAATATTTATTTAGACGACTCCCAAACAC and AGGGGACCACTTTGTACAAGAAAGCTGGGTGAGAAGTTAAATCAATGGAAATTAT-ACGCC by PCR and cloned via the Gateway system into the pDONR-zeo vector. The coding sequence for a *Strep*-*Tag-II* (Stratagene) was added to the cloned genomic *Su(var)2-1* construct using site directed mutagenesis with the primer pair AGTGACAAATGGCTTGGAGCCACCCGCAGTTCGAAAAAGATGAAAAAGAT and ATCTTT.

TTCATCTTTTTCGAACTGCGGGTGGCTCCAAGCCATTTGTCACT (Supplementary Table S2). The modified *Step*-*Tag-II* construct was cloned into a modified Gateway-converted pUAS-TattB-V5-3xFLAG vector (GenBank EF362409.1; Bischof et al. 2007) to obtain the tagged genomic rescue construct *Strep*-*Tag-II*-*Su(var)2-1*-*V5*-3x*FLAG*. All constructs were verified by DNA sequence analysis. Transgenic flies were generated using the *φ*C31-based integration into the *ZH*-*attP-51D* landing site (Bischof et al. 2007).

### CRISPR/Cas9-mediated HDR replacement of *Su(var)2-1*

The target DNA sequences selected for the CRISPR RNA-guided Cas9 nuclease were predicted using software (http://targetfinder.flycrispr.neuro.brown.edu/). The targeting sequence was cloned under the control of the *U6* promoter by annealing phosphorylated oligonucleotides to the pU6-BbsI-chiRNA plasmid at the *Bbs*I restriction sites. Donor templates containing *Su(var)2-1* homology arms (about 1 kb) were amplified by standard PCR methods and introduced into the pHD-DsRed vector. To generate the *Su(var)2-1* replacement donor pHD-DsRed^*Su(var)2-1*^, regions of homology flanking the S1 and S2 cleavage sites of around 1 kb in length were amplified (Phusion polymerase, Thermo Scientific) and incorporated via *EcoR*I and *Not*I restriction sites at the 5′- end and via *Pst*I and *Xho*I at the 3′- end (Supplementary Table S2) into the pHD-DsRed donor-vector (Gratz et al. 2014). In order to generate of targeting chiRNAs (Supplementary Table S2), the target-specific sequences for *Su(var)2-1* were synthesized as 19 bp-phosphorylated oligonucleotides, which were annealed and ligated into the *Bbs*I restriction sites of pU6-BbsI-chiRNA vector (Gratz et al. 2013). The pU6-BbsI-chiRNA vector containing the targeting gRNA (100 ng/μl) and the pHD-DsRed vector containing the donor templates (450 ng/μl) were co-injected as high-quality DNA into embryos produced by *M{vas-Cas9}ZH-2A/FM7c* flies, which express Cas9 in the germline. Positive *Su(var)2-1* knock-out lines were selected by screening for the DsRed marker.

### FISH analysis and immunohistochemistry

For FISH analysis of cycle 14 embryos after fixation with formaldehyde, the protocol of Phalke et al. (2009) was used with the following modifications: for preparation of digoxigenin-labeled probes, the desired sequence was amplified directly from the genomic DNA by using primers specific for the 359 bp satellite repeat, *Invader4* LTRs, the R1 element and the distal X chromosome respectively. Images were processed using the image software supplied (Zeiss, Germany). Primer sequences are listed in Supplementary Table S2.

### Polytene chromosome fixation and immunostaining

Salivary glands were dissected from 3rd instar larvae. Preparation of polytene chromosomes was performed as described previously (Silver et al. 1978) with the following modifications: salivary glands were dissected in 0.7% NaCl, fixed for 4 min and squashed in 55% (v/v) acetic acid/3% (v/v) formaldehyde. Chromosomes were incubated after blocking with 5% (w/v) skimmed milk powder in PBST (PBS with 0.05% Triton) with the indicated monoclonal or polyclonal antibodies (1 μg/ml) at 4 °C overnight, followed by incubation with fluorescently labeled secondary antibodies (1:250) for 2 h at 37°. For the list of antibodies, see Table S3. DNA of labeled preparation was stained with DAPI or Hoechst and mounted in VECTASHIELD antifade mounting medium. Preparations were examined with confocal laser-scanning microscopy (LSM 780, Zeiss) and processed with ZenPro software (Zeiss).

### Embryo fixation and immunostaining

*Drosophila* embryos were collected on apple juice agar plates, washed (0.7% w/v NaCl, 0.05% w/v Triton-X 100) into mesh baskets, and dechorinated in 12% (w/v) bleach for 2 min at room temperature. Dechorinated embryos were fixed with the boiling fix method as described (Rothwell and Sullivan 2000). Dechorinated and fixed embryos were then devitellenized in a 1:1 mixture of methanol–heptane. Dechorinated, fixed, devitellenized and dehydrated embryos were initially rehydrated in a series of increasing PBTA: methanol mixtures (PBS with 0.1% w/v Triton, 0.05% w/v BSA). After following rehydration in PBTA for 25 min embryos were then blocked in PBTA supplemented with 2% (w/v) skimmed milk powder and 3% (w/v) normal donkey serum for 1 h at room temperature. Prepared embryos were then incubated with the indicated primary antibodies (1 μg/ml) overnight at 4 °C in blocking buffer. Embryos were then washed three times with PBTA for 10 min each and then incubated with the appropriate fluorescently labeled secondary antibody (1:250) for 1 h in a dark room at 37 °C. Embryos were washed after incubation with secondary antibody again five times with PBTA for 10 min each. Hoechst-DNA-dye was added to the third wash. Finally, stained embryos were mounted on glass slides in VECTASHIELD antifade mounting medium or PBS supplemented with 50% (w/v) glycerol. Preparations were examined with confocal laser-scanning microscopy (LSM-780, Zeiss) and processed with ZenPro software (Zeiss).

### Ovary fixation and immunostaining

*Drosophila* ovaries of 2- or 3-day-old, well-fed female flies were dissected by hand in PBS buffer. The sheath surrounding the ovaries was removed and both pairs of ovaries were fixed with fixative (4% v/v paraformaldehyde) for 15 min. Ovaries were dissected and fixed with 4% (v/v) paraformaldehyde for 30 min at room temperature. The staining procedure was performed as described (Shcherbata et al. 2004) with the following modifications: after rinsing the ovaries with PBT (PBS/0.2% w/v Triton X-100) 3 times, they were incubated with methanol, rinsed again 3 times with PBT and then rehydrated for 1 h with PBT on a rotating wheel. Embryos and ovaries were incubated with primary antibodies (1:100/PBS + 1% w/v BSA + 0,05% w/v Triton X-100) overnight at 4 °C followed by incubation with Alexa Fluor 488 or 555-conjugated secondary antibody for 2 h at 37 °C (1:100/PBS + 1% w/v BSA + 0.05% w/v Triton X-100). Preparations were examined by confocal laser-scanning microscopy (LSM 510 and 780; Zeiss). Images were processed using the image software supplied by the microscope manufacturer (Zeiss, Germany). Antibodies used are listed in Supplementary Table S3.

### RT-PCR

Total RNA was extracted from larvae using TRIZOL™ reagent (Thermo Fisher Scientific) according to the user’s manual. An aliquot (1 μg) of extracted total RNA was used for cDNA synthesis using a first-strand cDNA synthesis kit (Promega). Equal amounts of cDNA samples were used in PCR reactions performed in triplicate in a standard PCR-cycler. Relative levels of mRNA were compared with the levels of *rp49* in each sample in a 1.0% (w/v) Agarose-Gel. Primers used in RT-PCR assays are listed in Supplementary Table S2.

### Chromatin immunoprecipitation

Fly heads were fixed with 1.8% (*v*/v) formaldehyde for 30 min at room temperature, homogenized, resuspended in RIPA buffer (140 mM NaCl, 10 mM Tris-HCl pH 8.0, 1 mM EDTA, 1% w/v Triton X-100, 0.1% w/v SDS, 0.1% w/v DOC). Staged embryo chromatin immunoprecipitation (ChIP) material (cycle 11 to cycle14) was prepared according to (Loubiere et al. 2017). Crosslinked material was sonicated after preparation in 4 ml of 10 mM Tris-HCl pH 8.0, 1 mM EDTA pH 8.0 for 30 min with a Branson 450 digital sonifier (45 cycles of 20 s on–40 s off). The sonicated lysate was clarified by centrifugation, preabsorbed by incubation with Dynabeads™ protein A magnetic beads (Thermo Fisher Scientific) and incubated with 7 μg polyclonal antibodies (α-H3K9ac, α-H3K27ac, α-H4K16ac) overnight at 4 °C. Antibody complexes were bound to protein A-Sepharose magnetic beads. Precipitated DNA was recovered and dissolved in 150 μl water. Control mock immunoprecipitations were done in parallel without antibodies. Real-time PCR analysis was performed according to previous studies (Dellino et al. 2004; Rudolph et al. 2007) and 5 μl DNA from each sample was amplified in 20 μl reactions with 2x SYBR Green Super Mix (Bio-Rad). All primer sequences used in the studies are listed in (Rudolph et al. 2007).

### Immunoprecipitation (GST-Trap) and immunoblotting

Salivary glands (100) were dissected in PBS solution and transferred in 300 μl of lysis buffer (20 mM HEPES pH 7,7; 1,5 mM MgCl_2_; 450 mM NaCl; 30 mM KCl; 0.25% w/v NP40; 0,1 mM EDTA; Roche protease inhibitor cocktail). Dissected glands were homogenized in the lysis buffer with an Eppendorf pestle and incubated at 4 °C on a rotating wheel for 30 min. Extracts were diluted after incubation by adding 600 μl dilution buffer (20 mM HEPES pH 7.7; 1.5 mM MgCl_2_; Roche protease inhibitor cocktail) and mixed for 5 min. Diluted extract was centrifuged at 4 °C and at 12300 rpm for 15 min to obtain the final salivary gland cell protein extract. Equilibrated GFP-Trap-Magnetic beads were incubated for 1 h at 4 °C on a rotating wheel with the salivary gland cell protein extract and afterwards washed 5 times with washing buffer (20 mM HEPES pH 7,7; 150 mM NaCl; 0.1% NP40; 0.15 mM EDTA; Roche Protease inhibitor cocktail). The immune complexes were washed with lysis buffer containing 500 mM NaCl five times (total 1 h) and subjected to immunoblot analysis with the indicated antibodies.

Chemicals, peptides, recombinant proteins, commercial assays and recombinant DNA used are listed in Supplementary Table S4.

### Phylogeny analysis

SU(VAR)2-1-like proteins of metazoans were collected using BLASTP based on the protein database and using tBLASTn based on the transcriptome shotgun assembly and the genome assembly database of NCBI. In part, the analyses were done locally using SU(VAR)2-1 protein sequences of the most closely related arthropod species. The orthology of the hits was evaluated by reciprocal BLAST. The resulting sequences were aligned by MUSCLE (Edgar 2004) using the Unipro UGENE interface, version 1.21 (Okonechnikov et al. 2012). A tree of selected proteins was built by RAxML online (https://raxml-ng.vital-it.ch/) using the substitution matrix LG und four gamma substitution rate categories. The resulting tree was re-rooted using Mesquite 3.40 (Maddison and Maddison 2018).

## Results

### *Su(var)2-1* encodes a new type of NRF1-domain protein with a C2HC ZnF motif

*Su(var)2-1* belongs to a group of *Su(var)* genes defined by butyrate/carnitine-sensitive mutations suggesting a function in control of histone deacetylation. The mutations are homozygous viable in females and semi-lethal in males but are lethal on media containing the inhibitors of histone deacetylation butyrate or carnitine (Reuter et al. 1982a; Dorn et al. 1986; Fanti et al. 1994). In addition, the mutations display lethal interaction with additional Y chromosome heterochromatin and are recessive female-sterile (Reuter et al. 1982a; Szabad et al. 1988; Dimitri and Pisano 1989). Crossover-mapping placed the gene near to the *Jammed* locus within chromosome region 31 on chromosome arm 2L. *Su(var)2-1* displays a haplo-dependent dominant Su(var) effect, which can be rescued by a duplication of the wild-type gene allowing duplication and deficiency mapping. Mapping crosses using a series of duplications generated by recombination between two inversions (Ryder et al. 2007) placed the *Su(var)2-1* gene to region 31A2-31B1. Deletion-mapping identified *CG5694* as *Su(var)2-1* (Fig. 1a). The P element *P[RS5]5-HA-1257* is inserted into the first intron of *Su(var)2-1* and causes aberrant splicing at the locus resulting in a *Su(var)2-1* mutant (Fig. S1a).

We identified 20 mutant alleles from different *Su(var)* mutant screens using complementation and rescue analysis. The alleles *2-1*^*210*^, *2-1*^*214*^ and *2-1*^*215*^ were independently isolated (Sinclair et al. 1992). According to the molecular lesions within the *Su(var)2-1* gene (Fig. 1b), a total of 15 of the 23 alleles are frame-shift or stop mutations. A hot-spot of four frame-shift mutations is found within a stretch of nine adenines that encode amino acid positions 346–349 (EKKT). Seven of the isolated alleles are point mutations. All of the frame-shift/stop alleles are agametic recessive female-sterile. Of the seven point mutations, the three alleles *2-1*^*03*^, *2-1*^*09*^ and *2-1*^*10*^ are female-fertile. The other point mutations (*2-1*^*08*^, *2-1*^*13*^, *2-1*^*14*^ and *2-1*^*16*^) are female-sterile but they may lay flaccid eggs without further development. The mutant effects were evaluated in trans-heterozygotes with the CRISPR/Cas9 generated *Su(var)2-1*^*ds*^ deletion of the locus (Fig. 1a). RT-PCR analysis showed no reduction of the *Su(var)2-1*-specific transcript in the six studied frame-shift alleles (*2-1*^*01*^, *2-1*^*02*^, *2-1*^*04*^, *2-1*^*05*^, *2-1*^*06*^ and *2-1*^*07*^) or the splice donor mutation (*2-1*^*04*^), thus excluding nonsense-mediated mRNA decay (Fig. S1b).

The SU(VAR)2-1 (CG5694) protein contains the C-terminal half of the NRF1/EWG (Nuclear Respiratory Factor-1/Erected Wing) domain at its N-terminus and a C2HC zinc-finger motif between amino acids 189 and 210 (Fig.1c). Two putative nuclear-targeting signals are found between amino acids 73–79 and 275–286. Mutations in the *Drosophila ewg* gene do not affect *white* gene silencing in *w*^*m4*^ (Fig. S2a). The SU(VAR)2-1 protein is conserved within insects, crustaceans and possibly also in some other Protostomata, but not in vertebrates (Fig. S2b), and shows homology with mammalian proteins through its NRF1 domain but not through its zinc-finger-containing region (Fig. S3a and S3b). Mouse Nrf1 (nuclear respiratory factor 1) is a close ortholog in mammals, which is a transcription factor whose binding is outcompeted by DNA methylation (Domcke et al. 2015).

### Female sterility and *Su(var)2-1* mutant rescue

We generated a series of transgenes for mutant rescue and expression of tagged SU(VAR)2-1 fusion proteins under endogenous promoter control. The *pP*{*UASTattB Strep-Su(var)2-1-V5-3xFLAG*} transgene produced a fusion protein carrying an N-terminal STREP and a C-terminal V5-3xFLAG tag and was placed under the control of the endogenous *Su(var)2-1* promoter. This construct rescued all *Su(var)2-1* mutant phenotypes, including the dominant Su(var) phenotype in the eyes of *In(1)w*^*m4h*^ flies (Fig. 1d) and all phenotypic defects observed in ovarian development of *Su(var)2-1* null females (Fig. S4a and Fig. S4c). *Su(var)2-1* null females only develop rudimentary ovaries with egg chambers that degenerate at stage 5–6 (Fig. S4b). Female sterility is follicle cell-dependent (Szabad et al. 1988). Typically, the number of follicle cells covering egg chambers is significantly reduced. During egg chamber development, SU(VAR)2-1 accumulates first in the prospective egg cell nucleus and becomes more abundant in nurse cell nuclei in older egg chambers (Fig. S4c). Effects of SU(VAR)2-1 on early embryogenesis could only be studied with the female-fertile point mutations *2-1*^*03*^, *2-1*^*09*^ and *2-1*^*10*^. However, these alleles showed all the characteristic mutant effects on chromatin organization like *Su(var)2-1* null alleles.

### SU(VAR)2-1 accumulates at heterochromatin in blastoderm nuclei

**Fig. 2:**
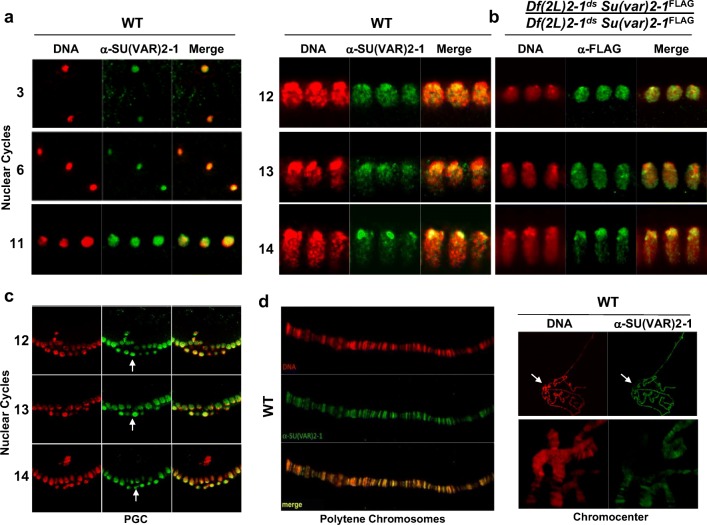
SU(VAR)2-1 is heterochromatin-associated in blastoderm nuclei but is a band-specific protein in polytene chromosomes. **a** SU(VAR)2-1 is an abundant chromatin protein in syncytial nuclei. At blastoderm cycles 11 to 14, the SU(VAR)2-1 protein preferentially associates with heterochromatin at the apical pole as shown for the endogenous protein (SU(VAR)2-1-specific polyclonal antibody) and in **b** for the STREP-SU(VAR)2-1-V5-3xFLAG fusion protein (monoclonal FLAG Antibody). **c** In contrast to somatic blastoderm cells where SU(VAR)2-1 is preferentially in prospective heterochromatin the protein shows uniform chromatin association in primordial germ line stem cell nuclei (arrow). **d** In larval salivary gland polytene chromosomes SU(VAR)2-1 is a band-specific protein and not found in chromocenter heterochromatin (arrows)

Chromatin association of SU(VAR)2-1 throughout development was studied with a specific polyclonal antibody generated against a peptide containing the 322 C-terminal amino acids and, additionally, by the *P*{*UASTattB Strep-Su(var)2-1-V5-3xFLAG*} transgene producing a STREP-SU(VAR)2-1-V5-3xFLAG fusion protein under endogenous promoter control (Fig. 2, Fig. S4c and S4d). In early cleavage, SU(VAR)2-1 is an abundant protein in syncytial nuclei. In blastoderm nuclei, polar Rabl organization of chromosomes is found with pericentric heterochromatin at the apical site and euchromatin toward the basal site (Foe et al. 1993; Rudolph et al. 2007). In early blastoderm, when heterochromatin and euchromatin formation is initiated, the SU(VAR)2-1 protein accumulated in pericentric heterochromatin at the apical site of blastoderm nuclei (Fig. 2a, b). In primordial germ-line cells, SU(VAR)2-1 was uniformly associated with chromatin (Fig. 2c) as in syncytial nuclei.

**Fig. 3:**
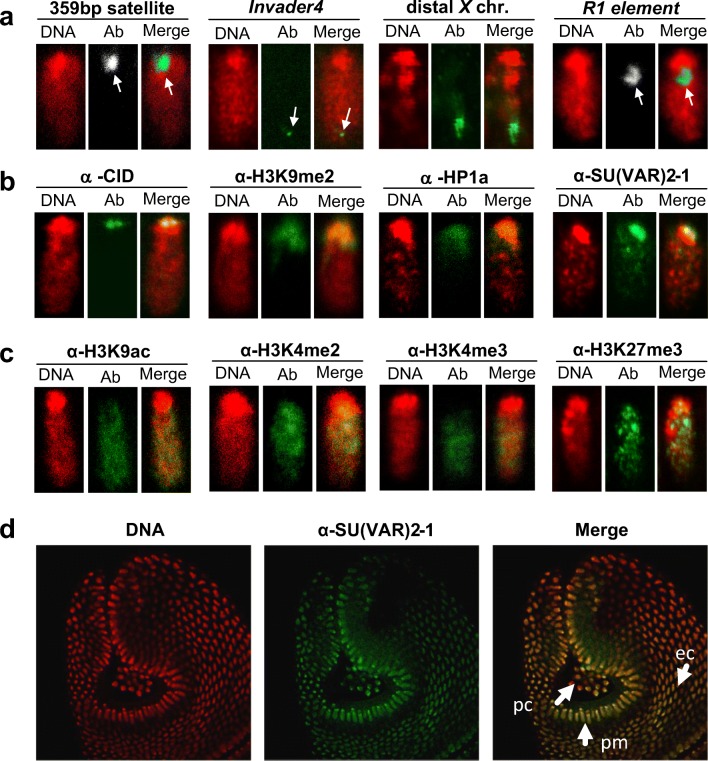
Apico-basal chromosome orientation and heterochromatin association of SU(VAR)2–1 in blastoderm nuclei. **a** Fluorescence in situ analysis with DIG-labeled DNA probes for the heterochromatic 359 bp satellite repeat, the sub-telomeric 2R and 3R *Invader4* repeats, a painting probe for the distal X chromosome and for the R1 retrotransposon repeat distal to the X-chromosomal nucleolus organizer. **b** Antibody staining for the centromere-specific protein CID, the heterochromatic H3K9me2 histone mark, the heterochromatin protein HP1a and SU(VAR)2-1. All the heterochromatic sequences, the heterochromatic histone marks and the HP1a and SU(VAR)2-1 proteins are apically located. **c** The euchromatic histone modification marks H3K9ac, H3K4me2, H3K4me3 and H3K27me3 identify euchromatin extending from heterochromatin toward the basal pole of the nuclei. **d** Embryo at early gastrulation showing the posterior midgut rudiment with internalized germ-line cells (glc). In nuclei of primordial cells in posterior midgut (pmg) SU(VAR)2-1 is rather uniformly distributed, although still more abundant at the apical pole of the nucleus. In nuclei of ectodermal cells (ec) SU(VAR)2-1 shows uniform nuclear distribution. DAPI staining of DNA in red, antibody and fluorescence staining in green

Heterochromatin association of SU(VAR)2-1 in blastoderm nuclei was confirmed by a study of apico-basal chromatin differentiation in blastoderm nuclei, which starts around cycle 11–13 (Fig. 3). Fluorescent in situ hybridization (FISH) with a probe specific for 359 bp satellite sequences labeled the apically located pericentromeric heterochromatin whereas a probe specific for the *Invader4* subtelomeric repeats of chromosome arms 2R and 3R identified the basally positioned telomeres (Fig. 3a). A painting probe for the distal 1A to 7A region of the X chromosome (Fuchs et al. 1998) further confirmed the suggested centromere-apical and telomere-basal orientation of chromosomes (Fig. 3a). A FISH probe for the non-LTR R1 retrotransposon, which forms a repeat cluster distal to the rDNA locus in the X chromosome (Tartof et al. 1984), marked the border region between heterochromatin and euchromatin (Fig. 3a). Staining for the centromere-specific protein CID showed the most apical positioning of centromeres in blastoderm nuclei (Fig. 3b). Immunostaining for H3K9me2 and the heterochromatin protein HP1a labeled the apically located pericentromeric heterochromatin (Fig. 3b). Euchromatic marks like H3K9ac, H3K4me2, H3K4me3 and H3K27me3 were uniformly spread from the border of heterochromatin toward the basal side of the nuclei (Fig. 3c).

The SU(VAR)2-1 protein in blastoderm nuclei was enriched, like the typical heterochromatic histone marks at the apically located pericentromeric heterochromatin (Fig. 3b), whereas in later embryogenesis during gastrulation SU(VAR)2-1 showed a rather uniform nuclear distribution (Fig. 3d).

Studies of larval polytene chromosomes revealed that SU(VAR)2-1 is found over bands and not in chromocenter heterochromatin (Fig. 2d). The developmentally specific heterochromatin accumulation of SU(VAR)2-1 in blastoderm nuclei when heterochromatin is established suggests that the protein is involved in initiation of heterochromatin formation during early embryogenesis. SU(VAR)2-1 is observed first in syncytial nuclei associated with all chromatin, then in blastoderm nuclei where it accumulated at pericentric heterochromatin. Later it leaves heterochromatin, and in polytene chromosomes binds the euchromatic bands and is excluded from the chromocenter (Fig. 2e). This suggests that SU(VAR)2-1 is mostly associated with euchromatin in somatic cell nuclei.

*Su(var)2-1* mutations display a strong dominant suppressor effect on all PEV rearrangements tested (Reuter et al. 1982b) and *w*^*m4h*^; *Su(var)2-1* mutant flies express a uniformly red-eye phenotype (Fig. 1d). The suppressor effect of *Su(var)2-1* mutations is as strong as a *Su(var)3-9* null mutations, which result in a complete loss of heterochromatin indexing by H3K9me2 (Schotta et al. 2002).

### SU(VAR)2-1 loss does not alter H3K9me2 but impairs the composite H3K9me2S10ph mark at pericentric heterochromatin

**Fig. 4:**
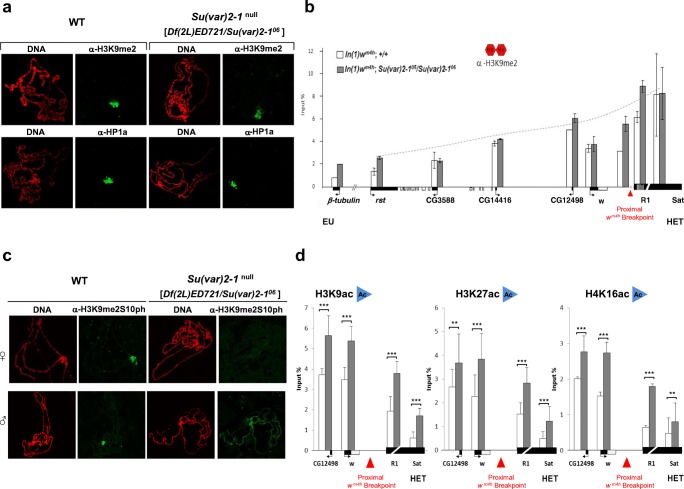
In *Su(var)2-1* null mutants heterochromatic H3K9me2- and HP1a-binding are unaffected, whereas H3K9me2S10pho double indexing is impaired. **a** Chromocenter staining for H3K9me2 and HP1a in larval salivary gland polytene chromosomes is identical between wild-type and a *Su(var)2-1* null {*Df(2L)ED721*/*Su(var)2-1*^*06*^} genotype. **b** ChIP analysis of H3K9me2 spreading along the *white*-*roughest* region juxtaposed in *In(1)w*^*m4h*^ to pericentric heterochromatin in adult female heads. No difference between wild-type (white bars) and *Su(var)2-1* null flies (gray bars) is found. Error bars indicate standard deviation. **c** Heterochromatin-specific double-indexing by H3K9me2S10pho is impaired in *Su(var)2-1* null larval salivary gland polytene chromosomes. In females, H3K9me2S10pho is lost, whereas it is ectopically distributed along euchromatic chromosome arms in the mutant males. **d** ChIP analysis of H3K9ac, H3K27ac and H4K16ac along the *white*-*roughest* region and in heterochromatin of *In(1)w*^*m4h*^ adult female heads. In the *Su(var)2-1* null genotype (white bars) elevated levels for all acetylation marks are found at euchromatin, at the R1 breakpoint sequences and for the heterochromatic 359 bp satellite sequences. Error bars indicate standard deviation. Statistical significance between the control and the mutant genotype with *P < 0.05, **P < 0.01 and ***P < 0.001. In B and C, the red triangle indicates the breakpoint of *In(1)w*^*m4h*^ in heterochromatin (HET), R1 indicates the retrotransposon cluster distal to the nucleolus organizer region and Sat the 359 bp satellite sequences in X chromosome heterochromatin

In *Su(var)2-1* mutant homozygotes, we examined immunocytologically H3K9me2 indexing of heterochromatin in larval salivary gland chromosomes and used ChIP analysis in adult heads. Interestingly, loss of a functional SU(VAR)2-1 protein did not interfere with H3K9me2 indexing of chromocenter heterochromatin in larval salivary gland polytene chromosomes (Fig. 4a). ChIP analysis of adult heads showed no reduction of H3K9me2 at the heterochromatic 359 bp satellite sequences and no reduction of H3K9me2 along the *white*-*roughest* euchromatic region juxtaposed to pericentric heterochromatin in *w*^*m4*^ (Fig. 4b). These results suggest that SU(VAR)2-1 functions independently of SU(VAR)3-9-dependent H3K9 di-methylation. This was further supported by immunostaining for HP1a, which showed normal chromocenter heterochromatin binding in larval salivary gland polytene chromosomes in *Su(var)2-1* null larvae (Fig. 4a). These results also show that H3K9me2 and HP1a are not alone sufficient to result in heterochromatic silencing.

Studies to determine the molecular basis for PEV-modifier effects displayed by *Jil1* mutations revealed that double H3K9me2S10ph indexing of pericentric heterochromatin is essential for *white* gene silencing in *w*^*m4*^ (Wang et al. 2014). These studies suggested that the histone H3S10-specific kinase JIL1 depends on the SU(VAR)3-9 H3K9 KMTase to establish the heterochromatin-specific composite H3K9me2S10ph mark in chromocenters of salivary gland polytene chromosomes (Wang et al. 2014).

In *Su(var)2-1* null mutants, indexing of heterochromatin with the composite H3K9me2S10ph histone mark was strongly impaired. In salivary gland polytene chromosomes of female larvae H3K9me2S10ph in chromocenter heterochromatin was strongly reduced, whereas in *Su(var)2-1* mutant male larvae significant ectopic distribution of the composite H3K9me2S10ph mark along the chromosomes was observed (Fig. 4c).

### *Su(var)2-1* null mutants gain global histone acetylation marks

Sensitivity of *Su(var)2-1* mutant homozygotes to inhibitors of histone deacetylase strongly suggests that histone deacetylation might be impaired. To examine this possibility, we first studied the levels of H3K9, H3K27 and H4K16 acetylation within the *w*^*m4h*^ PEV rearrangement by ChIP analysis in the heads of adult flies. In adult heads of *Su(var)2-1*^*05*^/*Su(var)2-1*^*06*^ females, all three acetylation marks were elevated in the tested heterochromatic as well as on the euchromatic sequences (Fig. 4d).

**Fig. 5:**
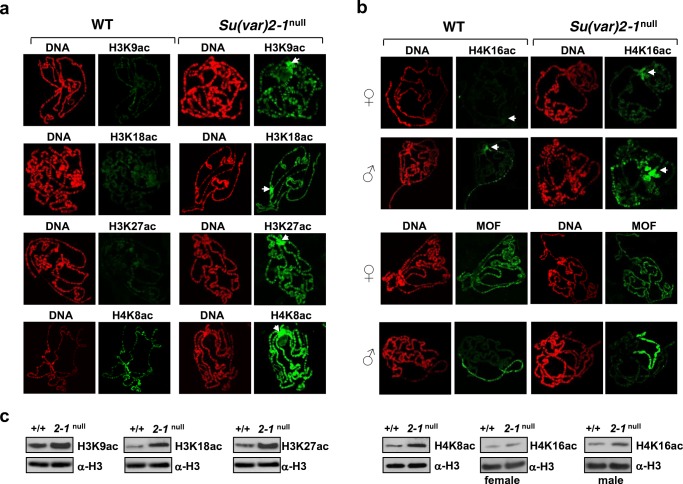
In *Su(var)2-1* null polytene chromosomes the levels of H3K9ac, H3K18ac, H3K27ac, H4K8ac and H4K16ac are strongly increased. **a** Antibody staining of *Su(var)2-1*^*06*^/*Df(2L)Su(var)2-1*^*ds*^ {*Su(var)2-1* null} larvae shows, when compared with wild-type, significantly higher levels of H3K9ac, H3K18ac, H3K27ac, H4K8ac and H4K16ac along the euchromatic chromosome arms and in chromocenter heterochromatin. White arrows point to chromocenters. **b** The increase in H4K16 acetylation is most prominent in chromocenter heterochromatin. In males the X-chromosome also shows increased staining for H4K16ac and MOF although no obvious effects on dosage compensation are observed. Compared with wild-type the male X-chromosome frequently appears to be more condensed in *Su(var)2-1* null mutant larvae. **c** The global increase in all the studied histone acetylation marks is supported by Western blot analysis

Next, we tested the levels of H3K9, H3K18, H3K23, H3K27, H4K5, H4K8, H4K12 and H4K16 acetylation on larval salivary gland chromosomes. Immunostaining and Western blot analysis in the *Su(var)2-1* null mutant showed a strong global increase in H3K9ac, H3K18ac, H3K27ac, H4K8ac and H4K16ac (Fig. 5a–c). Remarkably, larvae of the *Su(var)2-1* null mutant showed strong staining of chromocenter heterochromatin for H3K9ac, H3K18ac, H3K27ac, H4K8ac and H4K16ac (Fig. 5a, b). This suggests impaired indexing of heterochromatin with the silencing-associated H3K9me2 (Fig. 4a), the active H3K9ac and H3K27ac marks together with high levels of H4K16ac (Fig. 5a, b, Fig. S5a).

In addition, a significant increase in H4K16ac was found along autosomes in both females and males. Ectopic distribution and elevated levels of H4K16ac raises the question of whether a possible effect of the *Su(var)2-1* mutation on X-chromosome dosage-compensation occurs. We therefore studied chromosomal distribution of the MOF and MSL-1 components of the dosage-compensation complex DCC (Ferrari et al. 2014). Immunostaining with a MOF-specific antibody, when compared with wild-type, revealed no difference in chromosomal association of MOF in female and male larvae of the *Su(var)2–1* mutant. Specific association of MSL-1 with the male X-chromosome was also unaffected (Fig. S5a). H4K5ac, which is normally found in chromocenter heterochromatin, was not significantly changed in male *Su(var)2-1*-null larvae; however, in females, high H4K5ac-staining was only found in the chromocenter but appeared to be reduced along euchromatin (Fig. S5b).

The effects of *Su(var)2-1* overexpression on histone acetylation levels was studied for H3K9ac and H3K27ac in homozygous *Su(var)2-1*^+^*P*{*UAST-attB Strep-Su(var)2-1-V5-3xFLAG*} larvae carrying altogether four *Su(var)2-1*^+^ gene copies. Compared with wild-type larvae, both H3K9ac and H3K27ac were significantly reduced (Fig. S5c). In addition, *w*^*m4h*^ flies with four *Su(var)2-1*^+^ copies displayed a strong enhancement of white variegation in the eye (Fig. S5d). The data show that the dosage-dependent effect of *Su(var)2-1* on histone-acetylation levels was negatively correlated with its effect on heterochromatic gene silencing of the *white* gene in the *w*^*m4h*^ PEV rearrangement.

### SU(VAR)2-1 recruits the dRPD3 histone deacetylase to chromatin

**Fig. 6:**
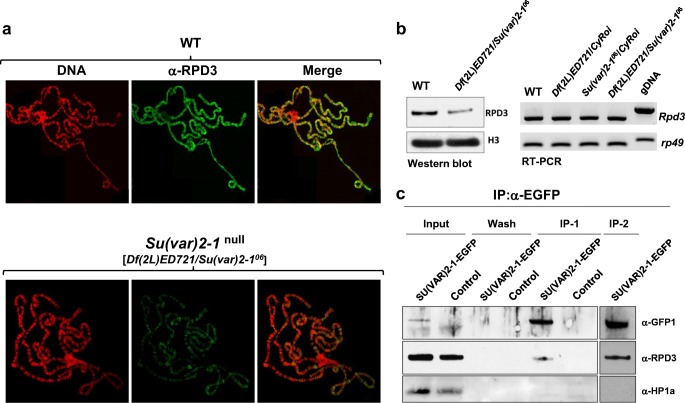
SU(VAR)2-1 recruits the histone deacetylase RPD3 to numerous chromosomal sites. **a** Immunostaining of *Su(var)2-1*-null larval salivary glands with a RPD3-specific polyclonal antibody shows significant reduction of RPD3 chromosome association. **b** Western analysis of *Su(var)2-1*-null [*Df(2L)ED721*/*Su(var)2-1*^*06*^] suggests global reduction of RPD3 although expression of the *Rpd3* gene is unchanged. **c** Co-immunoprecipitation of SU(VAR)2-1 and RPD3 was studied in extracts derived from transgenic larval salivary glands producing a SU(VAR)2-1-EGFP fusion protein purified with GFP-Trap beads. Precipitated proteins were studied by Western blot analysis using EGFP and RPD3 specific polyclonal antibodies. In Fig. 5c, the blots of two independent immunoprecipitations are shown (indicated with IP1 and IP2)

Due to the prominent role of the histone deacetylase dRPD3 (dHDAC1) in the control of gene expression during developmental processes (Chen et al. 1999; Miotto et al. 2006) through histone H3K9 and H3K27 deacetylation (Tie et al. 2009), it was important to study the nuclear distribution of RPD3 on larval salivary gland polytene chromosomes in *Su(var)2-1*-null mutant larvae. We identified a strong reduction in RPD3 chromosome-association (Fig. 6a), suggesting a pivotal role for SU(VAR)2-1 in RPD3 recruitment to chromosomes. Despite a reduced global level of the dRPD3 protein, the expression of the *dRpd3* gene was not affected by the *Su(var)2-1*-null genotype (Fig. 6b). The SU(VAR2-1-EGFP fusion protein was isolated using a GFP-Trap from larval salivary glands containing a *P{UAS*-*SU(VAR)2-1*-*EGFP}* transgene expressed by the *actin-GAL4* driver, and we tested by co-immunoprecipitation for its possible association with RPD3. The results showed significant association of RPD3 to SU(VAR)2-1 (Fig. 6c). These data suggest that the SU(VAR)2-1 protein is required for normal chromosomal association of RPD3.

### SU(VAR)2-1 controls chromatin restructuring before mid-blastula transition (pre-MBT)

**Fig. 7:**
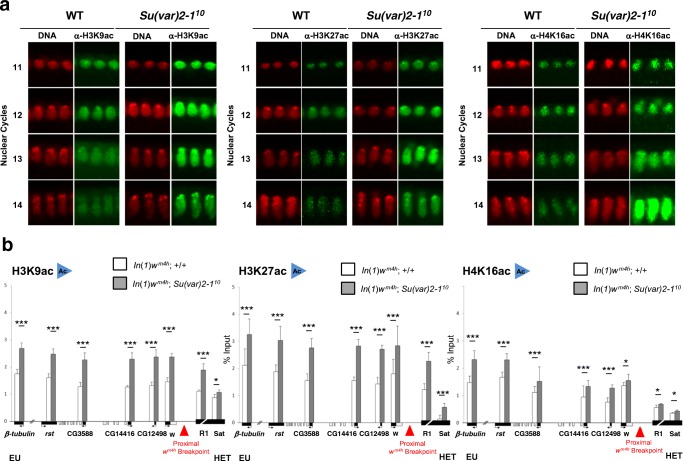
SU(VAR)2-1-controlled global histone deacetylation at pre-MBT is essential for normal heterochromatin formation. **a** Immunocytology of blastoderm nuclei at cycles 11, 12, 13 and 14 for H3K9ac, H3K27ac and H4K16ac from embryos produced by wild-type and *Su(var)2-1*^*10*^ homozygous females. In wild-type, the studied acetylation marks are prominent histone modifications in syncytial nuclei and in blastoderm at cycles 11 and 12 but are strongly reduced at cycles 13 and 14 when establishment of heterochromatin and euchromatin is initiated. Contrary to *Su(var)2-1*-null females, which are agametic homozygous, *Su(var)2-1*^*10*^ females are fertile. Requirement of SU(VAR)2-1 for histone deacetylation at pre-MBT is reflected by strong elevation of all studied acetylation marks in blastoderm nuclei of *Su(var)2-1*^*10*^ mutant embryos. **b** ChIP analysis of H3K9ac, H3K27ac and H4K16ac along the *white*-*roughest* region juxtaposed in the inversion *w*^*m4h*^ to pericentric heterochromatin (HET) in 0.5 h old wild-type (white bars) and *Su(var)2-1*^*10*^ homozygous embryos (gray bars). In *Su(var)2-1*^*10*^ mutant embryos the studied acetylation marks are elevated at all euchromatic sites. The heterochromatic R1 retrotransposon repeat cluster (marked by R1) at the proximal breakpoint of *In(1)w*^*m4h*^ (indicated by a red triangle) shows elevated levels of H3K9ac and H3K27ac whereas no significant differences are found for the 359 bp repeats (abbreviated Sat). However, all the studied heterochromatic sequences show substantial H3K9, H3K27 and H4K16 acetylation, indicating that the collected embryos include a considerable amount of cycle 11–14 embryos. Statistical significance between the control and the mutant genotype with *P < 0.05, **P < 0.01 and ***P < 0.001

In wild-type embryos abundant H3K9ac, H3K27ac and H4K16ac histone indexing was found up to nuclear cycle 12. Subsequent strong deacetylation of chromatin occurred between nuclear cycle 12 and 13 at pre-MBT in wild-type embryos (Fig. 7a). The SU(VAR)2-1/RPD3 interaction, which was demonstrated for salivary gland chromosomes, was also observed after co-immunoprecipitation experiments in early embryos (Fig. S6). In embryos produced by females that are homozygous for the hypomorphic *Su(var)2-1*^*10*^ allele, the abundant histone acetylation at pre-MBT was not removed by deacetylation (Fig. 7a). As a consequence, R1 heterochromatic sequences at the heterochromatic breakpoint of *w*^*m4h*^ showed elevated levels of H3K9ac and H3K27ac, as is observed for euchromatic X chromosomal sequences. At the heterochromatic 359 bp satellite sequences an increase in H3K27ac was found (Fig. 7b). Impairment of pre-MBT histone deacetylation in *Su(var)2-1*^*10*^ mutant embryos resulted in ambivalent histone modification at prospective heterochromatin, which was maintained and even intensified during consecutive development, as shown for adult heads (Fig. 4) and heterochromatic chromocenters at larval polytene chromosomes (Fig. 5). Together, these data suggest an essential role for SU(VAR)2-1 in induction of heterochromatin at pre-MBT.

### The SU(VAR)2-1 function at pre-MBT is maternally controlled

The maternal function of SU(VAR)2-1 in chromatin reorganization at pre-MBT was resolved by reciprocal crosses using a *P*{*UAST-attB Strep-Su(var)2-1-V5-3xFLAG*} transgene. The transgene expresses a SU(VAR)2-1 fusion protein with an N-terminal STREP and C-terminal V5-3xFLAG tag under the control of the endogenous *Su(var)2-1* promoter. This transgene effectively rescued all *Su(var)2-1* mutant phenotypes (Fig. 1d and Fig. S4). Using the SU(VAR)2-1 fusion protein, it was possible to monitor production of the SU(VAR)2-1 protein originating from the paternally inherited gene during embryonic development. The protein originating from the paternal allele was first detected in embryos at the beginning of gastrulation (Fig. S7), suggesting that all *Su(var)2-1* mutant phenotypes observed during early embryonic development depend on maternal contribution of SU(VAR)2-1. This can be concluded because a zygotic contribution of the paternal allele could only be detected at the beginning of gastrulation.

## Discussion

### Developmental regulation of step-wise heterochromatin establishment in *Drosophila*

*Su(var)* mutations of gene silencing in position-effect variegation (PEV) in *Drosophila* have been instrumental in the identification and functional analysis of chromatin components controlling establishment of heterochromatin. Histone H3K9 di- and tri-methylation is central to heterochromatin formation, which is catalyzed by the histone methyltransferases SU(VAR)3-9 in pericentric heterochromatin (Schotta et al. 2002) and dSETDB1 in the 4th chromosome, at telomeres, repeats and retrotransposons (Seum et al. 2007; Tzeng et al. 2007; Phalke et al. 2009). The H3K9me2 and H3K9me3 marks constitute a binding surface for the HP1a chromo domain (Fischle et al. 2003), which recruits a protein complex containing other *Su(var)* factors like dADD1 and SU(VAR)2-HP2 (Alekseyenko et al. 2014).

All these factors represent PEV *Su(var)* genes, which are essential in establishing a heterochromatic chromatin state. However, the heterochromatin-establishing SU(VAR) factors depend on the function of earlier-acting, heterochromatin-initiating SU(VAR) factors, which are required to generate pre-conditions for heterochromatin formation. dLSD1 {SU(VAR)3-3} was the first *Su(var)* gene to be identified that encodes a heterochromatin-initiating SU(VAR) factor, and which secures H3K9 methylation by the KMTase SU(VAR)3-9 (Rudolph et al. 2007). The dLSD1 histone demethylase binds preferentially to prospective heterochromatin in blastoderm nuclei and protects heterochromatic sequences against deposition of the active H3K4me1/me2 methylation marks in early MBT. SU(VAR)2-1 exerts a comparable function by controlling removal of abundant histone acetylation from prospective pericentric heterochromatin at pre-MBT through recruitment of the histone deacetylase RPD3.

More complex heterochromatin-initiating mechanisms have been revealed by new high-resolution techniques. TALE-light imaging showed H3K9me2/me3-independent HP1a recruitment to individual satellite sequences and the JabbaTrap technique revealed an important maternal function of the SETDB1 KMTase in heterochromatin initiation at early MBT (Yuan and O’Farrell 2016; Seller et al. 2019).

In *Schizosaccharomyces pombe*, the multi-enzyme complex SHREC containing the histone deacetylase Clr3 is essential for heterochromatin initiation (Sugiyama et al. 2007). In *Drosophila*, recruitment of RPD3 to prospective heterochromatin depends on SU(VAR)2-1, whereas in *S. pombe* recruitment of the SHREC complex is either Swi6/HP1-dependent or depends on sequence-specific binding proteins such as Atf1/Pcr1 (Yamada et al. 2005; Sugiyama et al. 2007). Establishment of facultative heterochromatin domains in *S. pombe* also depends on HDAC-dependent histone deacetylation (Watts et al. 2018). In *Arabidopsis thaliana*, initiation of heterochromatic silencing requires the histone deacetylase HDA6 (Aufsatz et al. 2002). Taken together, initiation of heterochromatic silencing by histone deacetylation may be a general and evolutionarily conserved mechanism in eukaryotes, whereas, due to the more complex developmental programs of higher eukaryotes, recruitment processes appear to differ significantly.

A third group of heterochromatin-maintaining SU(VAR) factors are also predicted and might protect the heterochromatic state for stable transmission across mitotic cell division. Although some of the heterochromatin-maintaining SU(VAR) factors are currently not fully defined, they are likely to comprise chromatin remodelers and/or histone-exchange factors. This is supported by the strong recessive suppressor effects of *acf1* mutations on *w*^*m4*^ PEV, a main component of the *Drosophila* ACF/CHRAC nucleosome remodeling complex (Fyodorov et al. 2004). In heterochromatin, nucleosomes are regularly spaced and their turnover is inhibited by the histone deacetylase Clr3, e.g. in fission yeast (Aygün et al. 2013). SU(VAR)2-1 in *Drosophila* might also have a role as a maintenance factor by recruiting RPD3 to many band regions, which are suggested to contain inactive genes.

### SU(VAR)2-1 and chromatin reorganization before mid-blastula transition (pre-MBT)

Syncytial nuclei in *Drosophila* divide by oscillating between DNA synthesis and mitosis without gap phases. Cell cycle control first occurs at cycle 13 by extension of the S-phase, when a G2-phase is introduced. Prolongation of the S-phase at cycle 13 is correlated with delayed replication of heterochromatic regions (McCleland et al. 2009; Shermoen and McCleland 2014; Yuan et al. 2014). The G1-phase is not introduced until cycle 17. Main zygotic genome activation occurs at mitotic cycle 14, which coincides with enrichment of active epigenetic marks, transcription and chromatin-remodeling factors (Darbo et al. 2013). Changes in cell cycle and zygotic genome activation represent the main well-studied events that define the period of MBT during early embryonic development in *Drosophila* (Yuan et al. 2016).

**Fig. 8:**
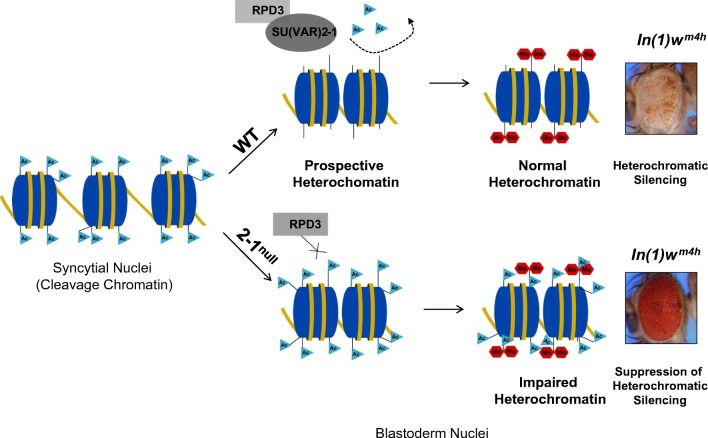
Heterochromatin formation depends on global histone deacetylation at the transition of naive syncytial (cleavage) chromatin to somatic blastoderm chromatin. SU(VAR)2-1 is an abundant chromatin protein of syncytial nuclei and in early blastoderm nuclei. At cycles 13–14 in blastoderm nuclei, SU(VAR)2-1 accumulates at heterochromatic regions at the apical pole. During transition of naive cleavage chromatin into somatic and germ-line chromatin, it is essential for global histone deacetylation to occur before mid-blastula transition. SU(VAR)2-1 is required for complete removal of, and protection against, histone acetylation at heterochromatic sequences. SU(VAR)2-1 physically interacts with RPD3 and is required for its normal chromatin association suggesting that RPD3 is the main deacetylase controlling early embryonic chromatin transition through H3K9ac, H3K27ac and H4K16ac deacetylation

However, global changes in chromatin organization at pre-MBT and the control of such processes had not yet been studied. Our data show that chromatin organization in the rapidly dividing syncytial nuclei is unique and differs significantly in the abundance of histone-indexing modifications. Abundant histone acetylation is found in the syncytial cleavage nuclei, whereas many other histone modification marks are under-represented or completely missing, including H3K4, H3K9, H3K27 methylation or H3K36me3 (Rudolph et al. 2007). SU(VAR)2-1 controls global histone deacetylation at pre-MBT, in particular for H3K9ac, H3K27ac and H4K16ac. This function of SU(VAR)2-1, by recruiting the RPD3 histone deacetylase, appears to be essential for transition of a cleavage chromatin state into an early blastoderm chromatin state, which is competent for differentiation of euchromatin and heterochromatin. Binding of SU(VAR)2-1 to heterochromatic sequences in blastoderm nuclei removes active histone acetylation marks from prospective heterochromatin by recruiting RPD3. Retaining high H3K9 and H3K27 acetylation levels in prospective heterochromatin in the blastoderm at pre-MBT causes impaired heterochromatin indexing, which later is maintained and even elevated during subsequent development (Fig. 8).

## Electronic supplementary material

ESM 1 **Fig. S1** The *Su(var)2-1 pP{RS5}5-HA-1257* insertional mutation forms an artificial chimeric splice product. **a** In *pP{RS5}5-HA-1257* the *Su(var)2-1* 5´UTR is spliced to the 3´ splice acceptor site of the second *white* gene exon resulting in a chimeric transcript of the *Su(var)2- 1* 5´UTR and the second exon of the *white* gene in *pP{RS5}5-HA-1257* homozygotes (RTPCR with primers Su1 and Su2). Flipase-induced recombination between the two FRT sites within *P{RS5}5-HA-1257* results in a loss of the second *white* gene exon with the 3´ splice acceptor site (remnant derivative *P{RS5r}5-HA-1257* element) reconstituting normal splicing to a *Su(var)2-1* wild-type transcript. RT-PCR analysis of *P{RS5}5-HA-1257*/+, *P{RS5}5-HA- 1257*/*P{RS5}5-HA-1257* and *P{RS5r}5-HA-1257*/*P{RS5r}5-HA-1257* genotypes using primers Su1 and Su2. In *P{RS5}5-HA-1257*/*P{RS5}5-HA-1257* homozygotes no *Su(var)2-1* transcript is detected whereas in *P{RS5r}5-HA-1257*/*P{RS5r}5-HA-1257* normal splicing occurs and the flies are Su+ with a wild-type mottled phenotype. **b** RT-PCR analysis of seven different frame-shift *Su(var)2-1* mutations excludes nonsense-mediated decay in all of the studied mutations. The *Su(var)2-1*^*03*^ point mutation was used as a control. In the heterozygotes of the *Su(var)2-1* frame-shift alleles *2-1*^*01*^, *2-1*^*02*^, *2-1*^*04*^, *2-1*^*05*^, *2-1*^*06*^ and *2-1*^*07*^ over *Df(2L)ED721* or *Df(2L)ED729* transcripts are detected using the for3 and rev4 primer pair flanking the mutant lesions in all the studied alleles. The stop mutation *2-102* with a 75 bp deletion shows a shorter transcript whereas in the *2-1*^*04*^ splice donor mutation a 67 bp larger transcript is formed containing intron IV. The other frame-shift alleles (*2-1*^*01*^, *2-1*^*05*^, *2-1*^*06*^ and *2-1*^*07*^) are all located within exon 5. **Fig. S2** Evolutionary conservation of *Su(var)2-1* in insects and crustaceans. **a** Mutations in the *erect wing* gene (*ewg*^*G687*^ and *ewg*^*2*^) do not modify *white* gene silencing in *w*^*m4h*^. **b** Maximum likelihood tree-build from selected SU(VAR)2-1 and NRF1/Erect Wing proteins. The selected proteins are representatives of different taxonomic groups identified by the species name. The *D. melanogaster* SU(VAR)2-1 protein is highlighted in bold (arrow). Normally-evolving SU(VAR)2-1 proteins are emphasized in black, rapidly-evolving SU(VAR)2-1 proteins in red (only identifiable using strongly related sequences, but supported by reciprocal BLAST), NRF1/Erect-Wing proteins are in blue and proteins with both conserved domains of SU(VAR)2-1 but without reciprocal BLAST support in green {SU(VAR)2-1-like proteins}. The scale below the tree presents amino acid replacements per site. **Fig. S3** Global alignment of the NRF1/EWG domain and the C2HC region of selected SU(VAR)2-1-related proteins built by MUSCLE. The grey number scale corresponds to the amino acid numbering in the alignment. The position of the first amino acid used is indicated before each protein sequence. *Drosophila melanogaster* SU(VAR)2-1 is in bold. The selected proteins are representatives of the taxonomic groups indicated after the species names. Conserved amino acid positions are marked in blue. **a** Alignment of the NRF1/EWG domain region of the selected SU(VAR)2-1 related proteins. **b** Alignment of the region containing the C2HC zinc-finger motif in SU(VAR)2-1 related proteins. **Fig. S4** Impairment of ovarian development in *Su(var)2-1* mutations and its rescue. **a** Loss of SU(VAR)2-1 results in rudimentary ovaries and females are agametic. Complete rescue of female sterility is observed in the presence of the *P*{*UAST-attB Strep-Su(var)2-1-V5- 3xFLAG*} transgene (indicated as *2-1*FLAG). *2-1*null abbreviates the *Df(2L)Su(var)2- 1ds*/*Su(var)2-106* trans-heterozygous genotype. **b** In *Su(var)2-1* null mutants ovary egg chamber development stops at stage 5-6. Mutant egg chambers become devoid of follicle cells. **c** All developmental defects in *Su(var)2-1* null ovarioles are rescued by *P*{*UAST-attB Strep-Su(var)2-1-V5-3xFLAG*}. The transgene is expressed under the control of the endogenous *Su(var)2-1* promoter and produces a SU(VAR)2-1 fusion protein with a Nterminal STREP and C-terminal V5-3xFLAG tag. **d** Western blot analysis of SU(VAR)2-1 in wild-type and mutant ovaries using a polyclonal SU(VAR)2-1 antibody detecting the endogenous protein or with a FLAG-specific antibody detecting the fusion protein expressed by the *P*{*UAST-attB Strep-Su(var)2-1-V5-3xFLAG*} transgene. **e** Abundant staining for SU(VAR)2-1 is found only in the oocyte nuclei suggesting that establishment of the naive cleavage type of chromatin is initiated early in oogenesis. **Fig. S5***Su(var)2-1* mutant effects on global H4K16 and H4K5 acetylation in heterochromatic chromocenters and SU(VAR)2-1 dosage effects. **a** Elevation of H4K16ac in chromocenters of *Su(var)2-1* null {*Df(2L)ED721*/*Su(var)2-1*^*06*^} female and male larvae. In wild-type, significant staining for H4K16ac within chromocenters is only found in male larvae. Loss of SU(VAR)2-1 causes a strong increase of H4K16ac in chromocenters of both females and males. **b** Immunostaining of H4K5ac in male larvae shows no significant difference between wild-type and *Su(var)2-1* null. In *Su(var)2-1* null female larvae strong chromocenter staining for H4K5ac is retained but along the euchromatic chromosome arms H4K5ac appears to be reduced. **c** Overexpression of *Su(var)2-1* by adding two additional genomic copies (4x*2-1*+) results in significant reduction of H3K9ac and H3K27ac staining at larval salivary gland chromosomes. **d** Dosage-dependent effects of SU(VAR)2-1 on *white* gene silencing in *w*^*m4h*^. Loss of one *Su(var)2-1* wild-type copy results in dominant suppression (haplo-insufficiency), whereas two additional copies (4x*2-1*^+^) result in strong enhancement of *white* gene silencing in *w*^*m4h*^. The dosage-dependent effects of *Su(var)2-1* on heterochromatic gene silencing in *w*^*m4h*^ are negatively correlated with the effects on histone deacetylation. **Fig. S6** Co-immunoprecipitation of SU(VAR)2-1 and RPD3 from 0-4h old embryos. Coimmunoprecipitation of SU(VAR)2-1 and RPD3 was studied in extracts derived from 0-4h old embryos produced by females homozygous for the *Df(2L)Su(var)2-1*^*ds*^*P*{*w+ UAS TattBStrep*- *Su(var)2-1*-V5-3x*FLAG*} chromosome. The transgene expresses the SU(VAR)2-1-V5- 3xFLAG fusion protein under the control of the endogenous *Su(var)2-1* promotor. The SU(VAR)2-1-V5-3xFLAG fusion protein was purified with α-FLAG-Trap beads. Precipitated proteins were studied by Western blot analysis using FLAG and RPD3 specific polyclonal antibodies. **Fig. S7** All embryonic SU(VAR)2-1 protein up to gastrulation is provided maternally. **a** For detection of maternal SU(VAR)2-1 and the protein originating from the paternally inherited gene we used the fusion protein encoded by the *P*{*UASTattB Strep-Su(var)2-1-V5-3xFLAG*} transgene (abbreviated 2-1^FLAG^). In genotypes with the *2-1*^*FLAG*^ transgene all endogenous *Su(var)2-1* genes are deleted by *Df(2L)Su(var)2-1*^*ds*^ (abbreviated *2-1*^*ds*^). **b** In cross 3, the tagged SU(VAR)2-1^FLAG^ fusion protein is first detected in embryos 2.0-2.5h after egg-laying. Western blots are shown of the SU(VAR)2-1^FLAG^ fusion protein using a monoclonal α-FLAG antibody. (PDF 2225 kb)

ESM 2 (PDF 332 kb)
