# Supplementary material for: Heterochromatin formation in Drosophila requires genome-wide histone deacetylation in cleavage chromatin before mid-blastula transition in early embryogenesis
Source: Chromosoma. 2020 Jan 16;129(1):83–98. doi: 10.1007/s00412-020-00732-x (PMC7021753; doi:10.1007/s00412-020-00732-x)
Supplement: Supplementary file 1 — Fig. S1 The Su(var)2-1 pP{RS5}5-HA-1257 insertional mutation forms an artificial chimeric splice product. a In pP{RS5}5-HA-1257 the Su(var)2-1 5´UTR is spliced to the 3´ splice acceptor site of the second white gene exon resulting in a chimeric transcript of the Su(var)2- 1 5´UTR and the second exon of the white gene in pP{RS5}5-HA-1257 homozygotes (RTPCR with primers Su1 and Su2). Flipase-induced recombination between the two FRT sites within P{RS5}5-HA-1257 results in a loss of the second white gene exon with the 3´ splice acceptor site (remnant derivative P{RS5r}5-HA-1257 element) reconstituting normal splicing to a Su(var)2-1 wild-type transcript. RT-PCR analysis of P{RS5}5-HA-1257/+, P{RS5}5-HA- 1257/P{RS5}5-HA-1257 and P{RS5r}5-HA-1257/P{RS5r}5-HA-1257 genotypes using primers Su1 and Su2. In P{RS5}5-HA-1257/P{RS5}5-HA-1257 homozygotes no Su(var)2-1 transcript is detected whereas in P{RS5r}5-HA-1257/P{RS5r}5-HA-1257 normal splicing occurs and the flies are Su+ with a wild-type mottled phenotype. b RT-PCR analysis of seven different frame-shift Su(var)2-1 mutations excludes nonsense-mediated decay in all of the studied mutations. The Su(var)2-103 point mutation was used as a control. In the heterozygotes of the Su(var)2-1 frame-shift alleles 2-101, 2-102, 2-104, 2-105, 2-106 and 2-107 over Df(2L)ED721 or Df(2L)ED729 transcripts are detected using the for3 and rev4 primer pair flanking the mutant lesions in all the studied alleles. The stop mutation 2-102 with a 75 bp deletion shows a shorter transcript whereas in the 2-104 splice donor mutation a 67 bp larger transcript is formed containing intron IV. The other frame-shift alleles (2-101, 2-105, 2-106 and 2-107) are all located within exon 5. Fig. S2 Evolutionary conservation of Su(var)2-1 in insects and crustaceans. a Mutations in the erect wing gene (ewgG687 and ewg2) do not modify white gene silencing in wm4h. b Maximum likelihood tree-build from selected SU(VAR)2-1 and NRF1/Erect Wing proteins. The selected [file 412_2020_732_MOESM1_ESM.pdf]

Figure S1

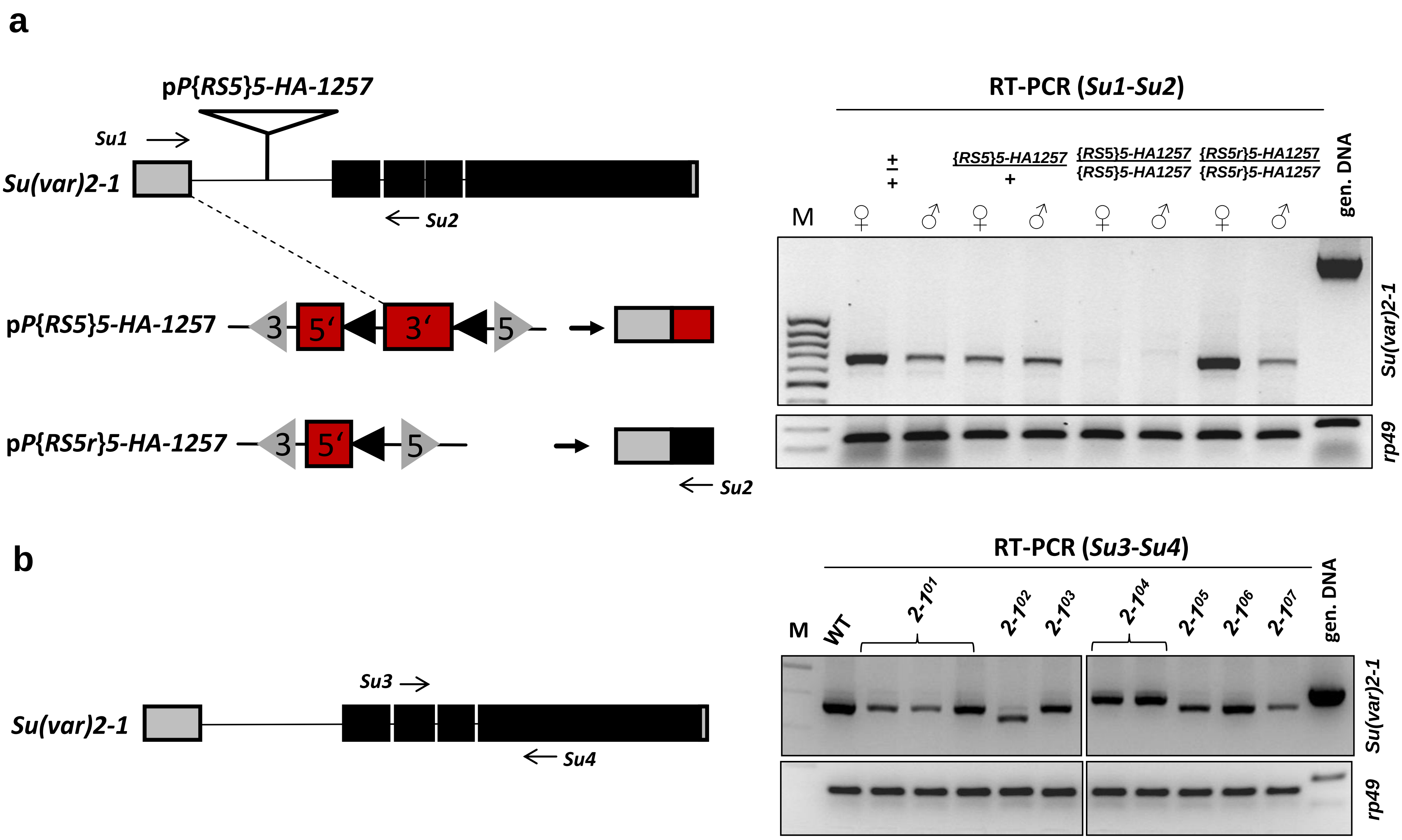
a
pP{RS5}5-HA-1257
Su1
Su(var)2-1
Su2
pP{RS5}5-HA-1257
3
5'
3'
5
pP{RS5r}5-HA-1257
3
5'
5
Su2
RT-PCR (Su1-Su2)
+
+
{RS5}5-HA1257
+
{RS5}5-HA1257
{RS5}5-HA1257
{RS5r}5-HA1257
{RS5r}5-HA1257
gen. DNA
M
♀
♂
♀
♂
♀
♂
♀
♂
Su(var)2-1
rp49
b
Su(var)2-1
Su3
Su4
RT-PCR (Su3-Su4)
M
WT
2-1^01
2-1^02
2-1^03
2-1^04
2-1^05
2-1^06
2-1^07
gen. DNA
Su(var)2-1
rp49

Figure S2

a

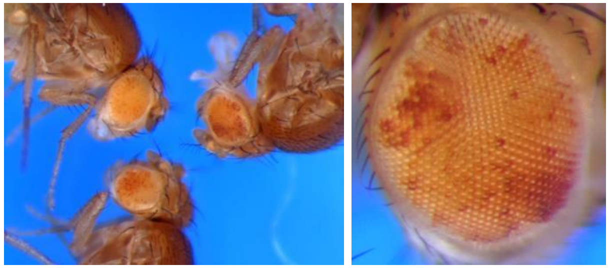

$w^{m4h}/ewg^{G687}\ w$

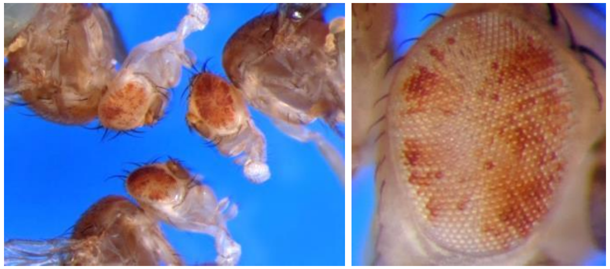

$w^{m4h}/ewg^{2}y^{1}w^{1}$

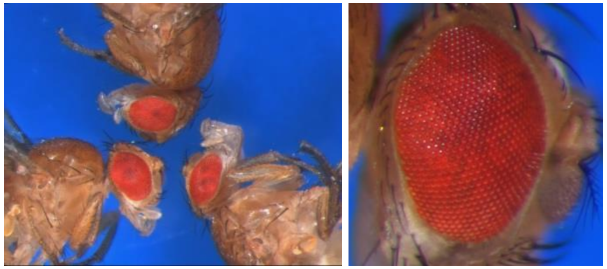

$w^{m4h};Su(var)2\text{-}1^{06}/CyO$

b

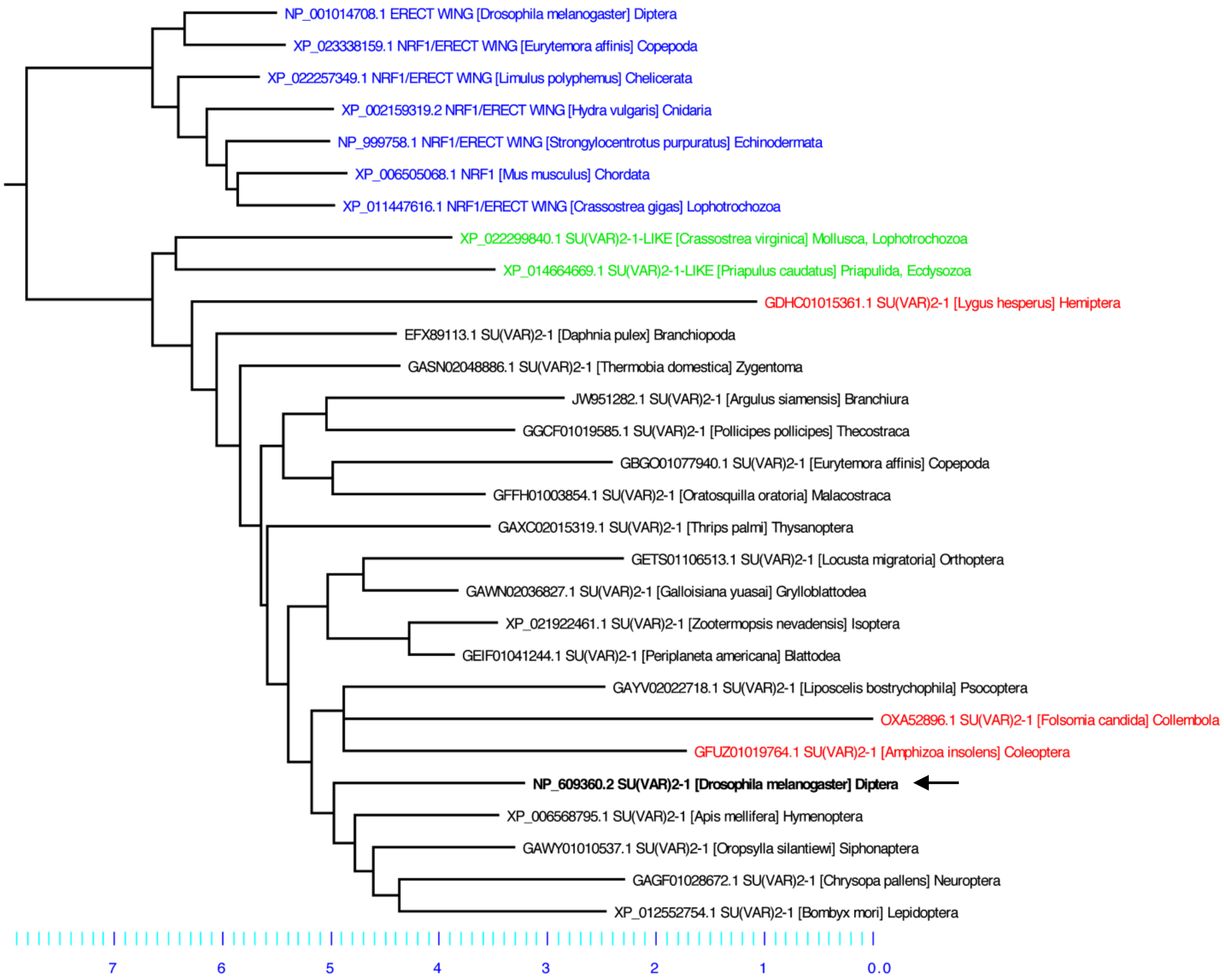

a

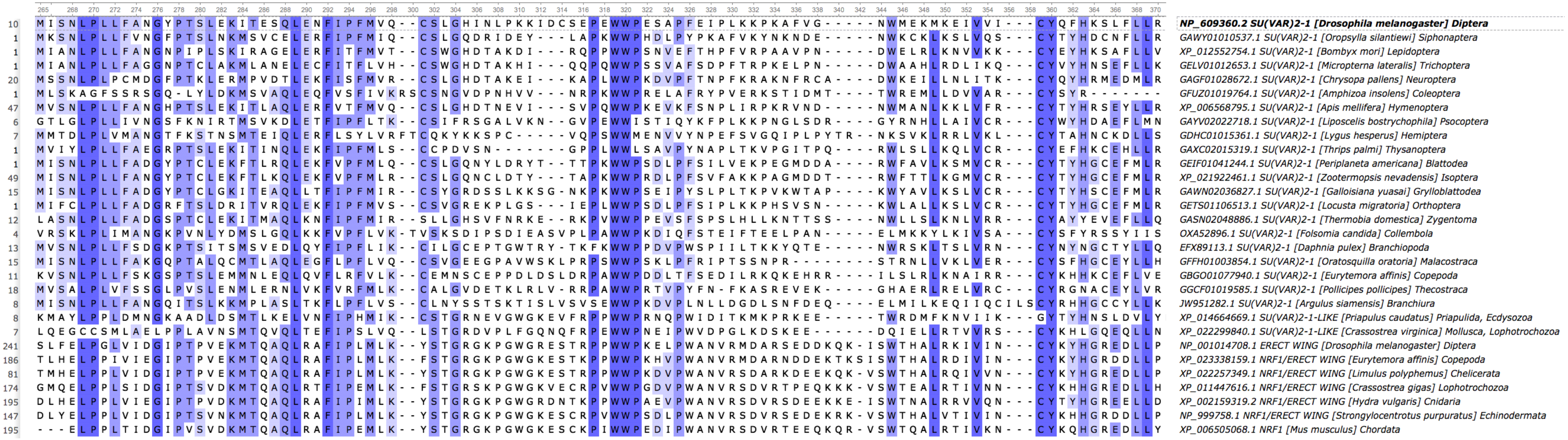
NP_609360.2 SU(VAR)2-1 [Drosophila melanogaster] Diptera
GAWY01010537.1 SU(VAR)2-1 [Oropsylla silantiewi] Siphonaptera
XP_012552754.1 SU(VAR)2-1 [Bombyx mori] Lepidoptera
GELV01012653.1 SU(VAR)2-1 [Micropterna lateralis] Trichoptera
GAGF01028672.1 SU(VAR)2-1 [Chrysopa pallens] Neuroptera
GFUZ01019764.1 SU(VAR)2-1 [Amphizoa insolens] Coleoptera
XP_006568795.1 SU(VAR)2-1 [Apis mellifera] Hymenoptera
GAYV02022718.1 SU(VAR)2-1 [Liposcelis bostrychophila] Psocoptera
GDHC01015361.1 SU(VAR)2-1 [Lygus hesperus] Hemiptera
GAXC02015319.1 SU(VAR)2-1 [Thrips palmi] Thysanoptera
GEIF01041244.1 SU(VAR)2-1 [Periplaneta americana] Blattodea
XP_021922461.1 SU(VAR)2-1 [Zootermopsis nevadensis] Isoptera
GAWN02036827.1 SU(VAR)2-1 [Galloisiana yuasai] Grylloblattodea
GETS01106513.1 SU(VAR)2-1 [Locusta migratoria] Orthoptera
GASN02048886.1 SU(VAR)2-1 [Thermobia domestica] Zygentoma
OXA52896.1 SU(VAR)2-1 [Folsomia candida] Collembola
EFX89113.1 SU(VAR)2-1 [Daphnia pulex] Branchiopoda
GFFH01003854.1 SU(VAR)2-1 [Oratosquilla oratoria] Malacostraca
GBGO01077940.1 SU(VAR)2-1 [Eurytemora affinis] Copepoda
GGCF01019585.1 SU(VAR)2-1 [Pollicipes pollicipes] Thecostraca
JW951282.1 SU(VAR)2-1 [Argulus siamensis] Branchiura
XP_014664669.1 SU(VAR)2-1-LIKE [Priapulus caudatus] Priapulida, Ecdysozoa
XP_022299840.1 SU(VAR)2-1-LIKE [Crassostrea virginica] Mollusca, Lophotrochozoa
NP_001014708.1 ERECT WING [Drosophila melanogaster] Diptera
XP_023338159.1 NRF1/ERECT WING [Eurytemora affinis] Copepoda
XP_022257349.1 NRF1/ERECT WING [Limulus polyphemus] Chelicerata
XP_011447616.1 NRF1/ERECT WING [Crassostrea gigas] Lophotrochozoa
XP_002159319.2 NRF1/ERECT WING [Hydra vulgaris] Cnidaria
NP_999758.1 NRF1/ERECT WING [Strongylocentrotus purpuratus] Echinodermata
XP_006505068.1 NRF1 [Mus musculus] Chordata

b

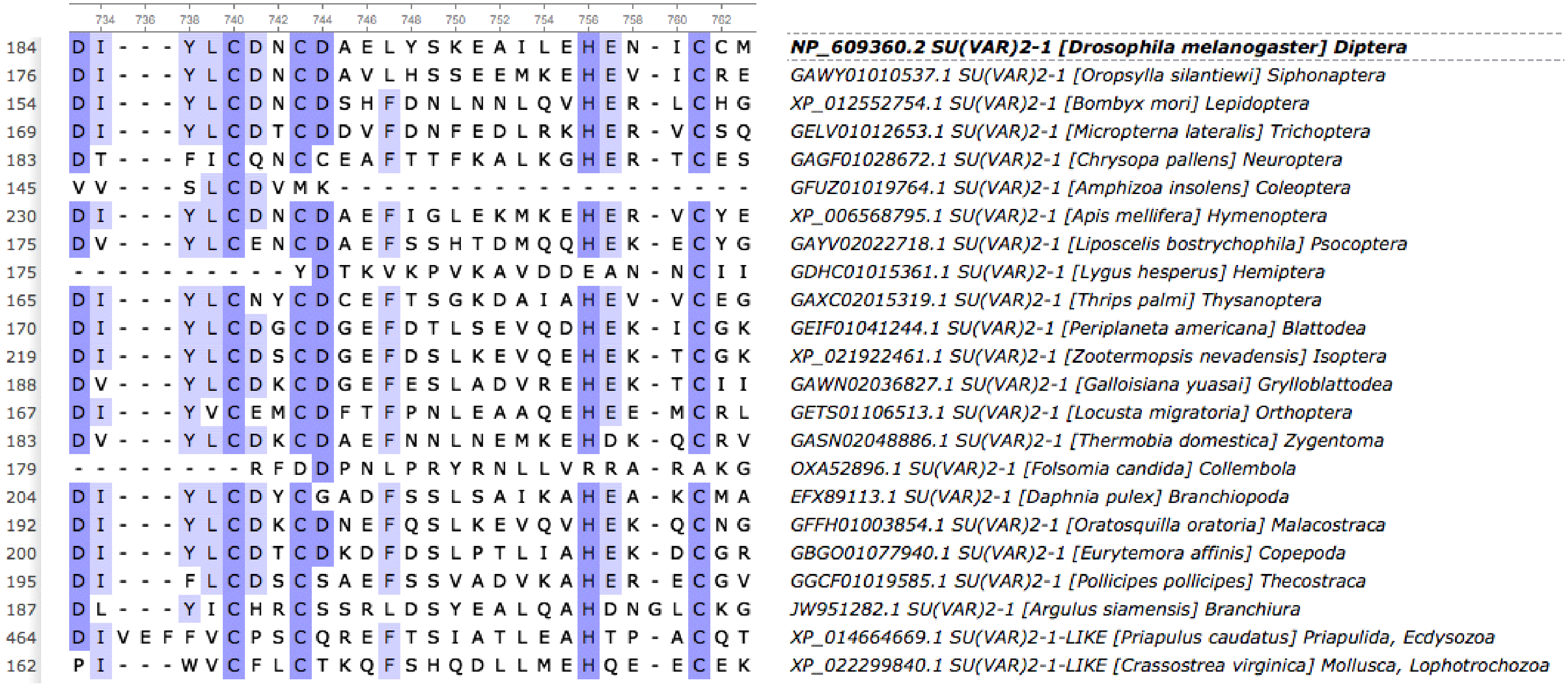
NP_609360.2 SU(VAR)2-1 [Drosophila melanogaster] Diptera
GAWY01010537.1 SU(VAR)2-1 [Oropsylla silantiewi] Siphonaptera
XP_012552754.1 SU(VAR)2-1 [Bombyx mori] Lepidoptera
GELV01012653.1 SU(VAR)2-1 [Micropterna lateralis] Trichoptera
GAGF01028672.1 SU(VAR)2-1 [Chrysopa pallens] Neuroptera
GFUZ01019764.1 SU(VAR)2-1 [Amphizoa insolens] Coleoptera
XP_006568795.1 SU(VAR)2-1 [Apis mellifera] Hymenoptera
GAYV02022718.1 SU(VAR)2-1 [Liposcelis bostrychophila] Psocoptera
GDHC01015361.1 SU(VAR)2-1 [Lygus hesperus] Hemiptera
GAXC02015319.1 SU(VAR)2-1 [Thrips palmi] Thysanoptera
GEIF01041244.1 SU(VAR)2-1 [Periplaneta americana] Blattodea
XP_021922461.1 SU(VAR)2-1 [Zootermopsis nevadensis] Isoptera
GAWN02036827.1 SU(VAR)2-1 [Galloisiana yuasai] Grylloblattodea
GETS01106513.1 SU(VAR)2-1 [Locusta migratoria] Orthoptera
GASN02048886.1 SU(VAR)2-1 [Thermobia domestica] Zygentoma
OXA52896.1 SU(VAR)2-1 [Folsomia candida] Collembola
EFX89113.1 SU(VAR)2-1 [Daphnia pulex] Branchiopoda
GFFH01003854.1 SU(VAR)2-1 [Oratosquilla oratoria] Malacostraca
GBGO01077940.1 SU(VAR)2-1 [Eurytemora affinis] Copepoda
GGCF01019585.1 SU(VAR)2-1 [Pollicipes pollicipes] Thecostraca
JW951282.1 SU(VAR)2-1 [Argulus siamensis] Branchiura
XP_014664669.1 SU(VAR)2-1-LIKE [Priapulus caudatus] Priapulida, Ecdysozoa
XP_022299840.1 SU(VAR)2-1-LIKE [Crassostrea virginica] Mollusca, Lophotrochozoa

Figure S4

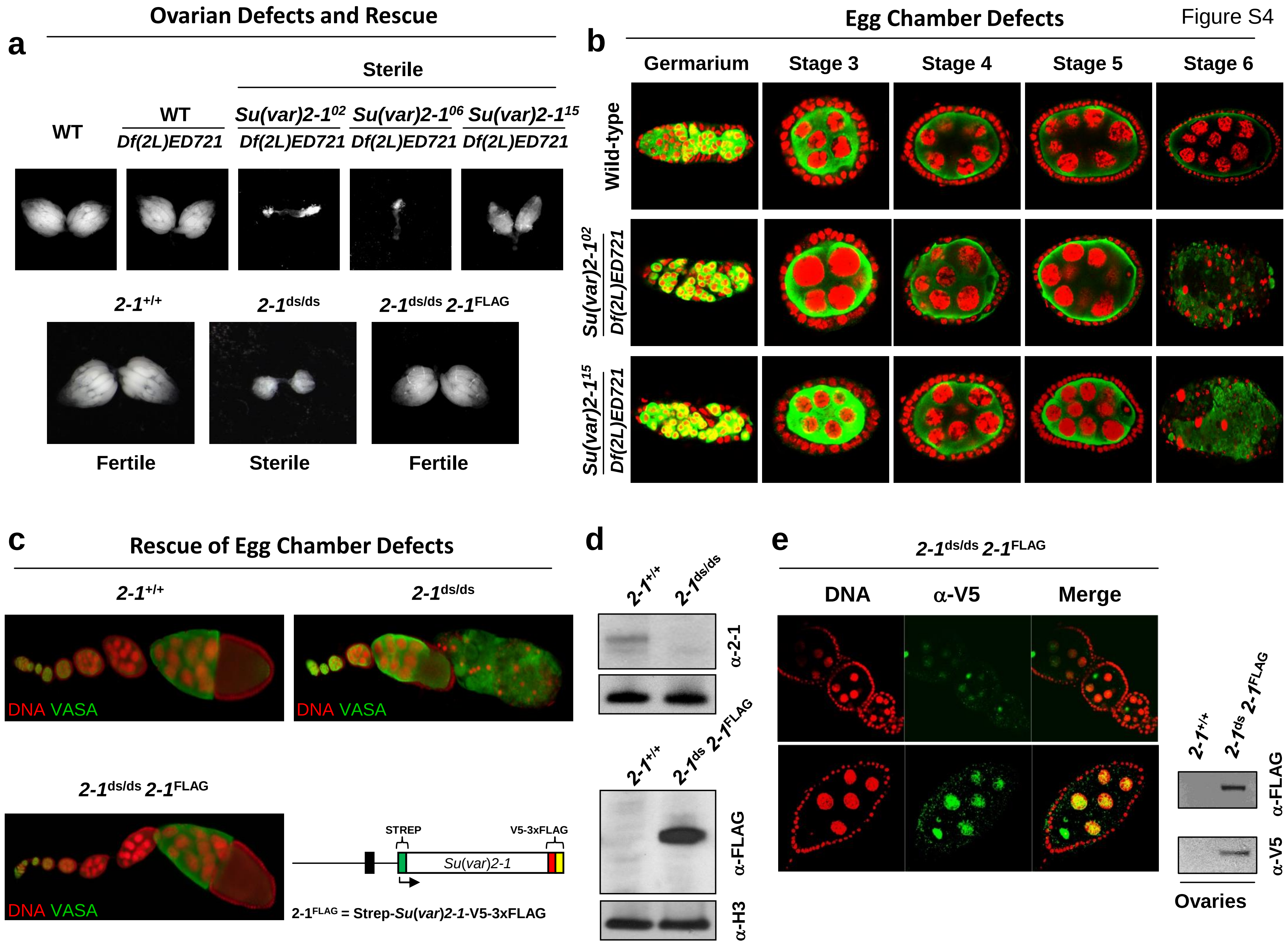

a
Ovarian Defects and Rescue
Sterile
WT
WT / Df(2L)ED721
$Su(var)2\text{-}1^{02}$ / Df(2L)ED721
$Su(var)2\text{-}1^{06}$ / Df(2L)ED721
$Su(var)2\text{-}1^{15}$ / Df(2L)ED721
$2\text{-}1^{+/+}$
$2\text{-}1^{ds/ds}$
$2\text{-}1^{ds/ds}$ $2\text{-}1^{FLAG}$
Fertile
Sterile
Fertile
b
Egg Chamber Defects
Germarium
Stage 3
Stage 4
Stage 5
Stage 6
Wild-type
$Su(var)2\text{-}1^{02}$ / Df(2L)ED721
$Su(var)2\text{-}1^{15}$ / Df(2L)ED721
c
Rescue of Egg Chamber Defects
$2\text{-}1^{+/+}$
$2\text{-}1^{ds/ds}$
DNA VASA
DNA VASA
$2\text{-}1^{ds/ds}$ $2\text{-}1^{FLAG}$
DNA VASA
STREP
V5-3xFLAG
Su(var)2-1
$2\text{-}1^{FLAG}$ = Strep-Su(var)2-1-V5-3xFLAG
d
$2\text{-}1^{+/+}$
$2\text{-}1^{ds/ds}$
α-2-1
$2\text{-}1^{+/+}$
$2\text{-}1^{ds}$ $2\text{-}1^{FLAG}$
α-FLAG
α-H3
e
$2\text{-}1^{ds/ds}$ $2\text{-}1^{FLAG}$
DNA
α-V5
Merge
$2\text{-}1^{+/+}$
$2\text{-}1^{ds}$ $2\text{-}1^{FLAG}$
α-FLAG
α-V5
Ovaries

Figure S5

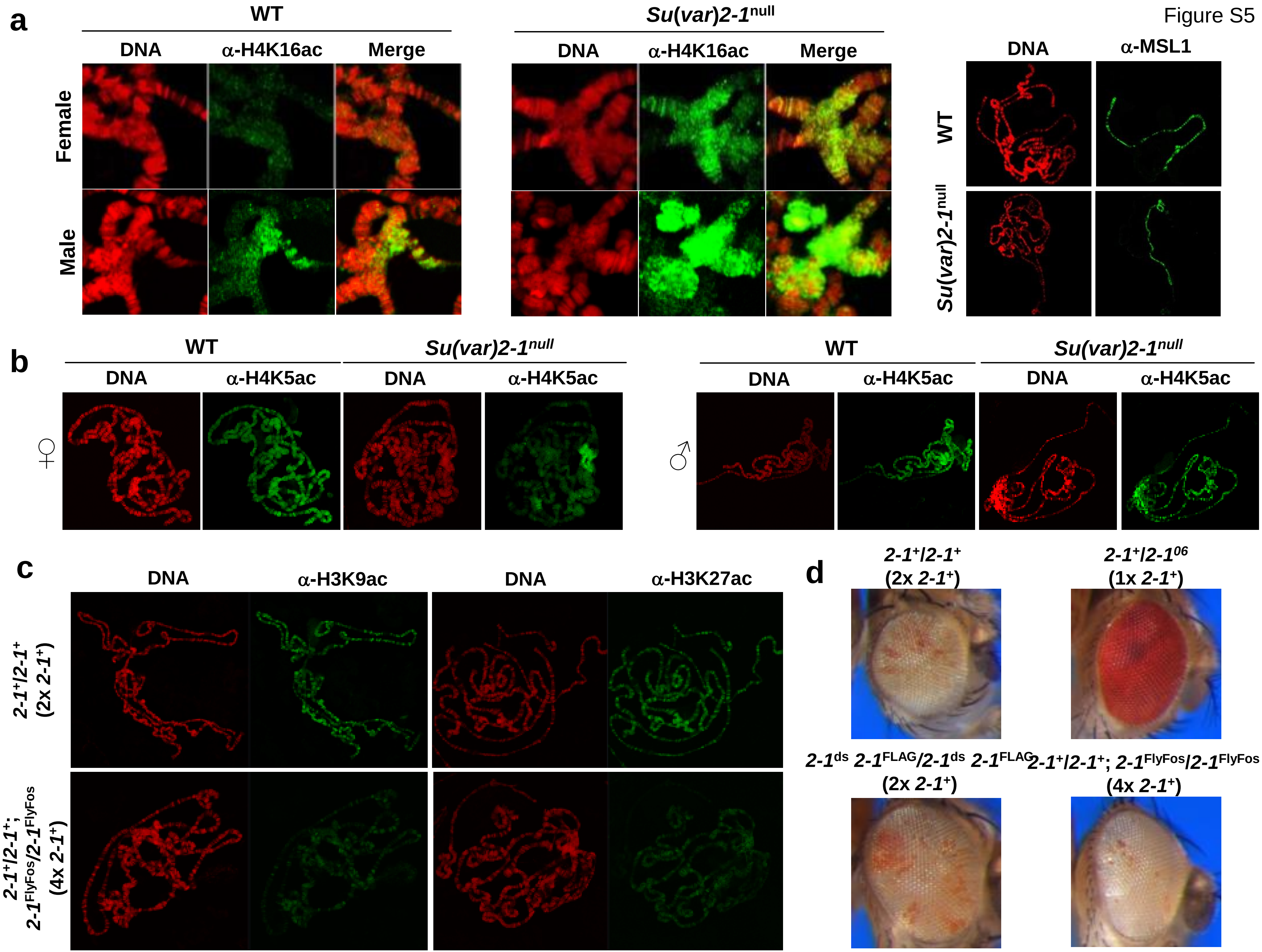
a
WT
DNA
α-H4K16ac
Merge
Su(var)2-1null
DNA
α-H4K16ac
Merge
Female
Male
DNA
α-MSL1
WT
Su(var)2-1null
b
WT
Su(var)2-1null
DNA
α-H4K5ac
DNA
α-H4K5ac
♀
WT
Su(var)2-1null
DNA
α-H4K5ac
DNA
α-H4K5ac
♂
c
DNA
α-H3K9ac
DNA
α-H3K27ac
2-1+/2-1+ (2x 2-1+)
2-1+/2-1+; 2-1FlyFos/2-1FlyFos (4x 2-1+)
d
2-1+/2-1+ (2x 2-1+)
2-1+/2-106 (1x 2-1+)
2-1ds 2-1FLAG/2-1ds 2-1FLAG (2x 2-1+)
2-1+/2-1+; 2-1FlyFos/2-1FlyFos (4x 2-1+)

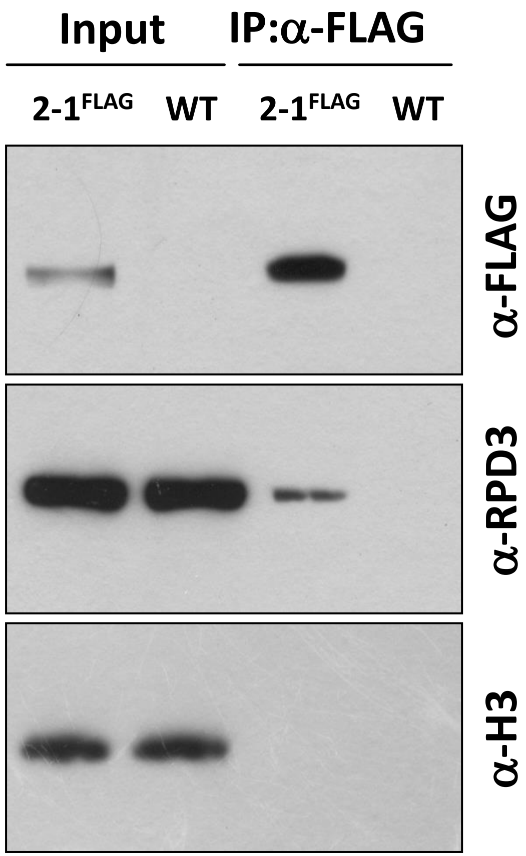

(0-4h old embryos)

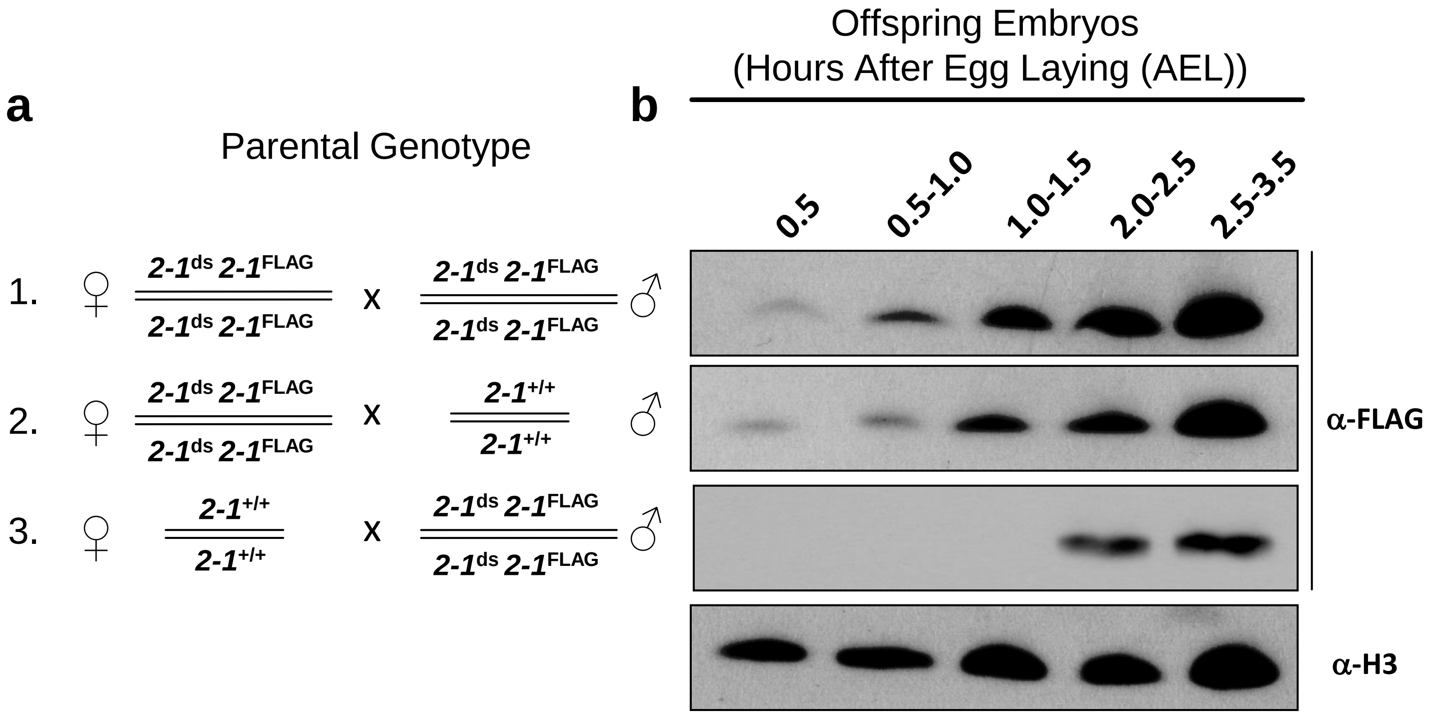
a
Parental Genotype
1. ♀ 2-1ds 2-1FLAG / 2-1ds 2-1FLAG x 2-1ds 2-1FLAG / 2-1ds 2-1FLAG ♂
2. ♀ 2-1ds 2-1FLAG / 2-1ds 2-1FLAG x 2-1+/+ / 2-1+/+ ♂
3. ♀ 2-1+/+ / 2-1+/+ x 2-1ds 2-1FLAG / 2-1ds 2-1FLAG ♂
b
Offspring Embryos
(Hours After Egg Laying (AEL))
0.5
0.5-1.0
1.0-1.5
2.0-2.5
2.5-3.5
α-FLAG
α-H3
