# Supplementary material for: Heterochromatin formation in Drosophila requires genome-wide histone deacetylation in cleavage chromatin before mid-blastula transition in early embryogenesis
Source: Chromosoma. 2020 Jan 16;129(1):83–98. doi: 10.1007/s00412-020-00732-x (PMC7021753; doi:10.1007/s00412-020-00732-x)
Supplement: Supplementary file 2 — (PDF 332 kb) [file 412_2020_732_MOESM2_ESM.pdf]

Supplementary Table S1 *Drosophila melanogaster* strains

Strains	Source	*Identifier/ Flybase ID*
In(1)w^{m4h}	G. Reuter lab stock	w^{m4h}
w^{1118}	Bloomington Stock Center	#3605
w^{m4h}; *P{UASp-Su(var)2-1-EGFP}/In(2L)t + In(2R)Cy*, *Cy Roi* cn^2 bw^{45a} or^{45a} sp^2	This paper	*Su(var)2-*1^{EGFP}
w^{m4h}; *P{UASp-attB-Strep-Su(var)2-1-V5-3xFLAG}/In(2L)t + In(2R)Cy*, *Cy Roi* cn^2 bw^{45a} or^{45a} sp^2	This paper	*Su(var)2-*1^{FLAG}
w^{m4h}; *Df(2L)Su(var)2-*1^{ds}*/In(2L)t + In(2R)Cy*, *Cy Roi* cn^2 bw^{45a} or^{45a} sp^2	This paper	*Su(var)2-*1^{ds}
w^{1118}; *Df(2L)BSC144/In(2LR)CyO*, $Duox^{Cy}$ dp^{lvI} pr^1 cn^2	Bloomington Stock Center	#9504 FBst0009504
w^{1118}; *Df(2L)BSC206/ In(2LR)CyO*, $Duox^{Cy}$ dp^{lvI} pr^1 cn^2	Bloomington Stock Center	#9633 FBst0009504
w^{m4h}; *P{FlyFos-026029-Su(var)2-1-V5-3xFLAG}/In(2L)t + In(2R)Cy*, *Cy Roi* cn^2 bw^{45a} or^{45a} sp^2	This paper	*2-*1^{FlyFos}
w^{1118}; *P{RS5}5-HA-1257/SM6*a, al^2 $Duox^{Cy}$ dp^{lvI} cn^{2P} sp^2	DrosDel	Ryder et al. 2007
w^{1118}; *P{RS5r}5-HA-1257/SM6*a, al^2 $Duox^{Cy}$ dp^{lvI} cn^{2P} sp^2	DrosDel (remnant element)	Ryder et al. 2007
w^{m4h}; *Df(2L)ED721/In(2L)t + In(2R)Cy*, *Cy Roi* cn^2 bw^{45a} or^{45a} sp^2	DrosDel; This Paper	Ryder et al. 2007
w^{m4h}; *Df(2L)ED729/In(2L)t + In(2R)Cy*, *Cy Roi* cn^2 bw^{45a} or^{45a} sp^2	DrosDel This Paper	Ryder et al. 2007
w^1; sna^{Sco}*/T(2;3)CyO*, *pP{*w^{+m} *hsp70*:*GAL4} pP{*w^{+m} *UAS*:*GFP} + TM3*, y^+ ri^1 p^p bx^{34e} e^s Ser^1 *pP{*w^{+m} *hsp70*:*GAL4} pP{*w^{+m} *UAS*:*GFP}/*Sb^1.	Reuter lab stock; Bloomington Stock Center	*T(2;3)CyOGFP-TM3GFP* #5703
w^{1118}; *P{*w^{+mC}*=Sgs3-GAL4.PD}TP1*	Bloomington Stock Center	#6870 FBst0006870
P{w^{+mC}*=EP}*ewg^{G687} *w**	Bloomington Stock Center	#27959 FBst0027959
ewg^2 y^1 w^1 sn^3*/FM6/Dp(1;Y)*y^2*61I*	Bloomington Stock Center	#38406 FBst0077893
w^{m4h}; *Su(var)2-*1^x*/In(2L)t + In(2R)Cy*, *Cy Roi* cn^2 bw^{45a} or^{45a} sp^2: *Su(var)2-*1^x = alleles *01*, *02*, *03*, *04*, *05*, *06*, *07*, *08*, *09*, *10*, *11*, *12*, *13*, *14*, *15*, *16*, *17*, *18*, *19* and *20* (all EMS induced)	This paper Figure 1	
w^{m4h}; *Su(var)2-*1^{210}*/In(2L)t + In(2R)Cy*, *Cy Roi* cn^2 bw^{45a} or^{45a} sp^2, w^{m4h}; *Su(var)2-*1^{214}*/In(2L)t + In(2R)Cy*, *Cy Roi* cn^2 bw^{45a} or^{45a} sp^2 and w^{m4h}; *Su(var)2-*1^{215}*/In(2L)t + In(2R)Cy*, *Cy Roi* cn^2 bw^{45a} or^{45a} sp^2	Sinclair et al., 1992	
ZH-attP-51D	Bischof et al. 2007	
y^1 *M{vas-int.Dm}ZH-2A w*; M{3xP3-RFP.attP}ZH-51D*	Bloomington Stock Center	#24483
y^1 *M{vas-Cas9}ZH-2A* w^{1118}*/FM7c*	Gatz et al. 2014	#51323

Supplementary Table S2 Oligonucleotide sequences

Primers for *Su(var)2-1* gRNA's design

1gRNA2_1sense	CTTC GAGGTACATACCGAAAAGAC
1gRNA2_1asens	AAAC GTCTTTTCGGTATGTACCTC
2gRNA2_1sense	CTTC GAATTCTTGGTGCGTACTGA
2gRNA2_1asens	AAAC TCAGTACGCACCAAGAATTC

Primers for homology arms of *Su(var)2-1*

CG5694-5' arm Pst F	TACTGCAGACAATTGGCTGAACAATACGATAAC
CG5694-5' arm Spe R	TAACTAGTGTACGCACCAAGAATTCGCAATG
CG5694-3' arm Eco F	TAGAATTCGTGTATCAAACTGCGGTCTTACGC
CG5694-3' arm Not R	TAGCGGCCGCTTTTCGGTATGTACCTCAACCCAAC

Construct - pP{UAST-attB Strep-Su(var)2-1-V5-3xFLAG}

*2-1*_genom_f_GW	GGGGACAAGTTTGTACAAAAAAG CAGGCTCAAAATATTTATTTAGACGACTCCCAAACAC
*2-1*_genom_r_GW	AGGGGACCACTTTGTACAAGAAA GCTGGGTGAGAAGTTAAATCAATGGAAATTATACGCC
*Strep1*_forw	AGTGACAAATGGCTTGGAGCCAC CCGCAGTTCGAAAAAGATGAAAAAGAT
*Strep2*_back	ATCTTTTTCATCTTTTTCGAACTGCGG GTGGCTCCAAGCCATTTGTCACT

Construct - pP[UAST cDNA-Su(var)2-1-EGFP]

Xho_*EGFP*_forw	ATACTCGAGTGTGAGCAAGGGCGAGGAGCTGTTCACC
Xho_*EGFP*_back	ATACTCGAGCTCGACTTGCCCGGGCCTTGTACAGC
*2-1*_cDNA-Eco	AAGAATTCATGGATGAAAAAGATAAAGCTCAA
*2-1*_cDNA-Not	ATGCGGCCGCCAGAAGTTAAATCAATGGAAATTA

RT-PCR

Su1	ATGGTGATTCTATTTTGATCGGTTTTT
Su2	AACCAACAGTTTGTTGTTTCTCCTGTCATA
Su3	GTGGTTATATGCTATCAATTTCACAAAAGC
Su4	GATATTGTTTGTTTTGTCGATTTTAACAATTGC
*rp49*_f	TGTCCTTCCAGCTTCAAGATGACCATC
*rp49*_r	CTTGGGCTTGCGCCATTTGTG
*Rpd3*_f2	TCAAATGTTGTTCTCCTTGGGGGC
*Rpd3*_b2	AGCGATTCCCGACGATGCCATCAAC

FISH analysis

359bp Sat for	ATAGGGATCGTTAGCACTGG
359bp Sat rev	ATTTTATTACGAGCTCAGTGAG
R1 for	CGAATGCTTGCCCGATATAC
R1 rev	GCACATCCATTTCATCCCC
Invader4 for	CACTGAAGGGATCTTCTTACATGTCC
Invader4 rev	TTCTGATGAAATTGTAATAATGCGGT

Supplementary Table S3 Antibodies

**Antibodies**	**Source**	**Identifier**
Rabbit polyclonal anti-H3	Abcam	Ab1791
Mouse monoclonal anti-H3K9me2	Abcam	Ab1220
Rabbit polyclonal anti-H3K9me2	T. Jenuwein (MPI-IE)	N/A
Mouse monoclonal anti-H3K9me2S10ph	Millipore/Merck Chemicals (Wang et al. 2014)	05-1354
Rabbit polyclonal anti-H3K9ac	Abcam	Ab10812
Rabbit polyclonal anti-H3K18ac	Abcam	Ab1191
Rabbit polyclonal anti-H3K27ac	Abcam	Ab4729
Rabbit polyclonal anti-H4K5ac	Millipore/Merck Chemicals	CS206839
Rabbit polyclonal anti-H4K8ac	Millipore/Merck Chemicals	CS205840
Rabbit polyclonal anti-H4K12ac	Millipore/Merck Chemicals	CS205842
Rabbit polyclonal anti-H4K16ac	Millipore/Merck Chemicals	CS205841
Rabbit polyclonal anti-H4K16ac	Santa Cruz	Sc-8662-R
Mouse monoclonal anti-FLAG	Sigma-Aldrich	F3165-5MG
Mouse monoclonal anti-HP1a	DSHB, Iowa USA	C1A9
Rabbit polyclonal anti-VASA	Santa Cruz Biotechnology	Sc-30210
Rabbit polyclonal anti-SU(VAR)2-1	This paper	N/A
Rabbit polyclonal anti-MOF	Gift from Peter Becker (LMU)	N/A
Rabbit polyclonal anti-RPD3	This paper	N/A
Goat polyclonal anti-GFP	TP Biolabs	TP401
Mouse monoclonal anti-V5	Thermo Fisher Scientific/Invitrogen	46-0705
AlexFlour^{TM} 488 goat anti-mouse	Thermo Fisher Scientific/Invitrogen	A11001
AlexFlour^{TM} 555 goat anti-mouse	Thermo Fisher Scientific/Invitrogen	A21422
AlexFlour^{TM} 488 goat anti-rabbit	Thermo Fisher Scientific/Invitrogen	A11008
Goat anti-rabbit HRP	Thermo Fisher Scientific/Invitrogen	G21234

Supplementary Table S4 Chemicals, peptides, recombinant proteins, commercial assays and recombinant DNA

**Chemicals, Peptides, and Recombinant Proteins**		
3xFLAG® Peptide	Sigma-Aldrich	F4799
Anti-FLAG® M2 Magnetic Beads	Millipore	M8823-1ML
cOmplete™ EDTA-free Protease Inhibitor Cocktail	Merk	5056489001
DAPI Solution	DB Bioscience	564907
GFP-Trap Magnetic Agarose	ChromoTek	gtm-20
Hoechst 33342	Sigma-Aldrich	B2261
Dynabeads™ Protein A for Immunoprecipitation	Thermo Fisher Scientific	10002D
VECTASHIELD® Antifade Mounting Media	Vector Laboratories	H-1200
**Commercial Assays**		
First strand cDNA synthesis Kit	Promega	A5003
NuPAGE™ 4-12% Bis-Tris Protein Gels	Thermo Fisher Scientific	NP0321BOX
QuantiTect Reverse Transcription Kit	Qiagen	205313
QuickChange II Site-Directed Mutagenesis Kit	Agilent Technologies	200524
**Recombinant DNA**		
pP{UAST-attB}	Bischof et al. 2007	DGRC#1419*
pP{UAST-attB-Strep-Su(var)2-1-V5-3xFLAG}	This paper	N/A
pP{UAST}	Brand and Perrimon 1993	DGRC#1000*
pP{UAST-Su(var)2-1-EGFP}	This paper	N/A
pP{FlyFos}	Ejsmont et al. 2009	N/A
pP{Su(var)2-1^{FlyFos}}	This paper	FlyFos #026029
pHD-DsRed	Gift of Melissa Harrison, Kate O'Connor-Giles and Jill Wildonger	Addgene #51434
pU6-BbsI-chiRNA	Gratz et al. 2013	Addgene #45946

*Drosophila Genomic Resource Center (NIH Grant 2P40OD0100949)
